# High immunogenicity of virus-like particles (VLPs) decorated with *Aeromonas salmonicida* VapA antigen in rainbow trout

**DOI:** 10.3389/fimmu.2023.1139206

**Published:** 2023-05-22

**Authors:** Jeong In Yang, Dagoberto Sepúlveda, Irina Vardia, Jakob Skov, Louise Goksøyr, Adam F. Sander, Niels Lorenzen

**Affiliations:** ^1^ National Institute of Aquatic Resources, Technical University of Denmark, Kongens Lyngby, Denmark; ^2^ Centre for Medical Parasitology, Department of Immunology and Microbiology, University of Copenhagen, Copenhagen, Denmark; ^3^ AdaptVac Aps, Copenhagen, Denmark

**Keywords:** Virus-like particles (VLPs), fish diseases, *A. salmonicida*, furunculosis, aquaculture, recombinant vaccine

## Abstract

The Gram-negative bacterium *A. salmonicida* is the causal agent of furunculosis and used to be one of the most loss-causing bacterial infections in the salmonid aquaculture industry with a mortality rate of about 90% until the 1990s, when an inactivated vaccine with mineral oil as adjuvant was successfully implemented to control the disease. However, the use of this vaccine is associated with inflammatory side effects in the peritoneal cavity as well as autoimmune reactions in Atlantic salmon, and incomplete protection has been reported in rainbow trout. We here aimed at developing and testing a recombinant alternative vaccine based on virus-like particles (VLPs) decorated with VapA, the key structural surface protein in the outer A-layer of *A. salmonicida*. The VLP carrier was based on either the capsid protein of a fish nodavirus, namely red grouper nervous necrotic virus (RGNNV) or the capsid protein of *Acinetobacter phage* AP205. The VapA and capsid proteins were expressed individually in *E. coli* and VapA was fused to auto-assembled VLPs using the SpyTag/SpyCatcher technology. Rainbow trout were vaccinated/immunized with the VapA-VLP vaccines by intraperitoneal injection and were challenged with *A. salmonicida* 7 weeks later. The VLP vaccines provided protection comparable to that of a bacterin-based vaccine and antibody response analysis demonstrated that vaccinated fish mounted a strong VapA-specific antibody response. To our knowledge, this is the first demonstration of the potential use of antigen-decorated VLPs for vaccination against a bacterial disease in salmonids.

## 1 Introduction

Vaccines based on recombinant structural viral proteins auto-assembling into virus-like particles (VLPs) have been used for more than a decade for protecting humans against diseases caused by viruses such as hepatitis B virus and human papillomavirus (1). Since VLPs do not carry any viral genome inside, there is no risk of transmitting disease, and the virus-like symmetric and repetitive structure has appeared to provide high immunogenicity (1, 2). This makes VLPs attractive vaccine candidates as alternatives to traditional killed or attenuated vaccines as well as to other recombinant vaccines, which often retain low immunogenicity. Although no VLP-based vaccines have yet been licensed for aquaculture, there is considerable experimental evidence of the potential of this type of vaccine for protecting fish against viral diseases like infectious pancreatic necrosis (IPN), viral nervous necrosis (VNN), and pancreatic disease (PD) (3). We thus recently demonstrated how vaccination of sea bass with VLPs auto-assembled in yeast (*Pichia pastoris*) cells expressing the RGNNV capsid protein (Cp) induced a strong antibody response along with long-lasting protection against VNN (4). One limiting parameter is that use of VLP-based vaccines in their native form is only possible for diseases caused by viruses, and essentially viruses for which production of VLPs is relatively straight forward requiring only a single or a few structural viral proteins. However, experiments with fusing N-or C-terminal peptides of different sizes to the recombinantly expressed viral capsid proteins have demonstrated that adding short peptides do not disturb the CP assembly into VLPs. Particularly for the RGNNV Cp, C-terminal peptide fusion was shown to result in peptide exposure on the VLP surface (5). Although such peptide-covered VLPs are likely able to trigger an antibody response to the peptide, it will often be desirable to decorate the VLPs with larger antigens to obtain a protective immune response. The earlier developed SpyTag/SpyCatcher split-protein technology for covalently coupling of two proteins was recently shown to be applicable for decorating VLPs with vaccine antigens (6, 7).

We earlier reported SpyTag/SpyCatcher-based coupling of *Streptococcus inia* α-enolase on the surface of RGNNV VLPs, and preliminary vaccination trials in olive flounder and zebrafish indicated a certain level of protection (8). In this study, we aimed at testing the antigen-decorated VLP vaccine concept against furunculosis, a septicemic disease in rainbow trout caused by the Gram-negative bacterium *Aeromonas salmonicida* (9). Although commercial bacterin-based oil-adjuvanted vaccines against furunculosis are available, insufficient protection implicating needs for treatment with antibiotics regularly occurs in sea-reared rainbow trout (10). This calls for alternative vaccines. Apart from the LPS-coat characteristic for Gram-negative bacteria, virulent forms of *A. salmonicida* have an outer A-layer mainly composed of the VapA protein (11). We here report the generation of recombinant VapA fused to SpyCatcher and coupling to VLPs based on SpyTag-fused Cp of either RGNNV or AP205 followed by immunization/vaccination of rainbow trout and analysis of antibody response profiles and protection against furunculosis.

## 2 Materials and methods

### 2.1 VLP production and purification

The VLP-SpyTag cassette encoding the SpyTag peptide fused to the C-terminus of RGNNV capsid protein was previously constructed in the pET28a+ vector (8). Following plasmid amplification in *E. coli* Top10 (ThermoFisher), the plasmid was transformed into *E. coli* BL21(DE3) (ThermoFisher) for protein expression. Starting from a single colony, transformed bacteria were grown in a horizontal shaker (200rpm) at 37°C in LB broth containing 50μg/ml of kanamycin until the optical density at 600 nm (OD_600_) reached 0.4-0.5 corresponding to approx. 5 × 10^8^ cfu/ml. At this point, IPTG corresponding to 0.1mM was added followed by continued incubation for 18 h at 20°C. The OD_600_ hereby reached 1.3-1.5, and the cells were collected by centrifugation at 5000 × g for 15 min. The pellet was resuspended in Tris-HCl buffer (0.5M NaCl, 20mM Tris-HCl, pH6.9) and sonicated at an amplitude of 15 microns (corresponding to 2 × 10^13^ Hz) with 8 pulsations for 30 s (Soniprep 150, MSE) on ice. Following centrifugation at 16000 × g for 10 min. the supernatant of the bacterial sonicate was collected and filtered through a 0.45μm Millipore Ultra free sterile centrifugal unit before loading on the top of a 25% sucrose cushion. After ultracentrifugation at 112700 × g (SW28 rotor, Beckman) for 2 h at 4°C, the supernatant was discharged and the pellet was resuspended in Tris-HCl buffer (pH6.9) and centrifuged at 16000 × g for 10 min at 4°C to remove non-dissolved aggregates. The protein concentration was measured using bicinchoninic acid assay (BCA) kit (ThermoFisher) with BSA as reference according to the manufacturer’s instructions. RGNNV VLPs without the SpyTag were produced according to the same procedure. The AP205 VLPs with and without SpyTag were prepared as described earlier (12).

### 2.2 Preparation of the VapA-SpyCatcher antigen

To clone VapA gene ORF of *A. salmonicida*, genomic DNA was isolated from *A. salmonicida* 090710-1/23 grown in 5ml TB broth at 20°C. The VapA gene was amplified using VapA_NdeI_F and VapA_L_R as forward and reverse primers (**Table 1**) respectively, in a PCR reaction including 0.5pmol primers, 200ng template DNA, and 1 × GoTaq^®^ G2 Colorless Master Mix (Promega) and 30 cycles of 30 s at 95°C, 30 s at 58°C, and 72°C 90 s in a Bio-Rad thermocycler. The amplified VapA gene was loaded on a 0.7% agarose gel and purified by a gel extraction kit (Qiagen). SpyCatcher was amplified using L_SpyCatcher_F and SpyCatcher_XhoI_R primers and purified similarly. Subsequently, VapA was C-terminally connected to SpyCatcher by overlapping PCR using VapA_NdeI_F and SpyCatcher_XhoI_R primers (**Table 1**) and inserted into the pCR2.1+TOPO vector (Invitrogen). The VapA-SpyCatcher cassette was subsequently transferred into pET28a+ using the terminal NdeI and XhoI restriction sites (**Supplementary Figure 3**). The VapA-SpyCatcher construct was transformed into *E. coli* BL21(DE3), which was first grown at 37°C in LB broth containing kanamycin (50μg/ml) to OD_600 =_ 0.5-0.6, and then induced with 0.1 or 0.5mM IPTG for 3 h at 37°C, 5 h at 27°C, or 18 h at 20°C. After spinning 5000 × g for 10 min, the bacterial pellets were suspended in Tris-HCl buffer (pH 6.9) and lysed by sonication. Then, the lysed bacteria were centrifuged at 16000 × g for 15 min at 4°C and the supernatant was collected into another tube. Pellets and supernatants were loaded on SDS-PAGE gels to examine the levels of VapA-SpyCatcher protein expression and aggregation.

**Table 1:** Primers used in this study.

Primers	Sequence (5’–3’)
**VapA_NdeI_F**	CATATGATGTTTAAGAAGACTTTGATTGCAGC
**VapA_L_R**	ACCGGAACCCCCTGAACCCAGAGTGAAATCTACCA
**L_SpyCatcher_F**	GGTTCAGGGGGTTCCGGTGGCGCCATGGTTGATACCTTAT
**SpyCatcher_XhoI_R**	CTCGAGAATATGAGCGTCACCTTTAGTTGCT

Underline, restriction enzyme.

After optimizing the culture conditions, bacteria expressing VapA-SpyCatcher were grown at 20°C for 18 h after induction with 0.1mM IPTG, and then harvested, sonicated and centrifuged as above. The supernatant was further cleared by 0.45μm syringe-filtration before loading on a nickel-based agarose (Ni-NTA) His bind resin chromatographic column (Novagen). After rinsing with washing buffer (0.5M NaCl, 60mM imidazole, 20mM Tris-HCl, pH 6.9), the bound protein was eluted using elution buffer (0.5M NaCl, 0.5M imidazole, 20mM Tris-HCl, pH 6.9) and fractions of 1ml were collected. The protein concentration in each fraction was determined using the BCA kit. After pooling the peak fractions, imidazole was removed by dialysis using Slide-A-Lyzer 10K MWCO MINI dialysis device (ThermoFisher).

### 2.3 Coupling of VapA-SpyCatcher to VLPs

To display the VapA antigen on the VLP surface, purified RGNNV VLP-SpyTag or AP205 VLP-SpyTag were mixed at 1:1 or 2:1 molar ratios with VapA-SpyCatcher, and incubated at 4°C for 2 h or 24 h (12). These reactions were performed in Tris-HCl buffer (pH 6.9). Aliquots of the mixtures were subsequently centrifuged at 16000 × g for 2 min. Pre- and post-spin samples were then loaded on SDS-PAGE gels for examination of coupling reactions and to examine for precipitation/formation of aggregates. Following staining with Coomassie blue, the relative amounts of RGNNV and AP205 Cps were estimated by densitometric analysis of the Cp bands. Coupling efficiencies were calculated by comparing densitometry data before and after the coupling reaction using ImageJ software (13) (Version 1.53o).

All protein samples, including vaccines, were examined for stability following freezing and thawing by three cycles of snap freezing on dry ice. Samples were centrifuged at 16000 × g for 2 min after each thawing and aliquots were examined by SDS-PAGE and densitometric analysis.

### 2.4 Electron microscopy

To examine VLP shape and size, 5µl of VLPs diluted to 0.2mg/ml was applied on 300-mesh cupper carbon film grids (AGS160-3, Agar scientific, England) followed by absorption of excessive buffer using filter paper (Whatman #1) after 2 min, and negative staining with 5µl of 2% uranyl acetate for 1 min following standard procedures. Imaging was done using a Tecnai T12 (BioTwin, Canada) microscope at 120kV. Images were recorded with a camera TemCam-XF416 (TVIPS, Gauting, Germany). The samples were subsequently examined by transmission electron microscopy (TEM) using FEI Tecnai T12 BioTwin. Image settings: Conventional/basic TEM, Accelerating voltage 120kV, Condenser aperture 150µm, Magnification 30000X. Exposure time for image aquisition: 3-5 seconds.

### 2.5 Dynamic light scattering

VLPs with the concentration of 0.4 – 0.5 mg/ml were centrifuged at 16000 × g for 2 min at 4°C to discard any aggregates. Then, 100μl of each VLP sample was loaded in cuvettes, mounted in the DLS chamber, and measured at 25°C to determine particle size distribution profile using a DynaPro NanoStar DLS instrument equipped with a 658nm laser (WYATT Technology). The VLPs were measured in triplicate with 20 acquisitions of 5 s and particle populations evaluated. Percent polydispersity (%Pd) was calculated using Wyatt DYNAMICS software.

### 2.6 Vaccine preparations

Conjugations were performed with either RGNNV VLP-SpyTag or AP205 VLP-SpyTag and VapA-SpyCatcher by incubation for 24 h at 4°C in a 1:1 molar ratio. Excessive VapA-SpyCatcher was removed either by dialysis overnight against Tris-HCl buffer (pH6.9) using a 1000 MWCO dialysis tubing (Spectrum) or by pelleting the decorated VLPs by ultracentrifugation at 250000 × g (SW 50.1 rotor, Beckman) for 180 min. followed by resuspension in Tris-HCl buffer (pH6.9). Protein concentration was determined using the BCA kit. Solutions of purified VLP-SpyTag – VapA-SpyCatcher conjugates (VLP-VapA) contained approximately 400 µg protein/ml, and the samples were examined by SDS-PAGE to confirm removal of uncoupled VapA-SpyCatcher. In contrast to mammals, fish are not sensitive to *E. coli* endotoxin/LPS and we therefore did not make an effort to completely eliminate nor to quantify LPS in our VLP preparations.

For the preparation of formalin-killed cell (FKC) as a bacterin-reference vaccine, *A. salmonicida* strain 090710-1/23 was grown in TB broth for 48 h at 20°C. When the bacterial culture reached OD600 of 1.5, cells were pelleted at 2500 × g and resuspended in an equal volume of PBS. Before inactivation, the CFU count was 5 × 10^9^/ml. Formalin inactivation was performed by adding formaldehyde to a final concentration of 0.7% followed by overnight incubation at 4°C. Subsequent to washing and resuspension in PBS, sterility was confirmed by plating on blood agar plates.

Vaccines were diluted in Tris-HCl buffer to the desired final concentration. These were adjusted to obtain similar amounts of VapA antigen in VLP-VapA groups within each vaccination Trial (see below) and administered with or without emulsification in equal volumes of Freund’s Incomplete adjuvant (FIA) (Sigma-Aldrich).

### 2.7 Vaccination and challenge trial-1

Rainbow trout (13 ± 2g), hatched and reared under pathogen-free conditions, were maintained at 12°C in 180 L tanks supplied with air and running tap water. Fish were anesthetized in water with benzocaine (0.01%) and vaccinated intraperitoneally (IP) with 100µl of RGNNV VLP-VapA vaccine (22µg), AP205 VLP-VapA vaccine (17µg), formalin-killed *A. salmonicida* (corresponding to 10^6^ CFU), or Tris-HCl buffer (control). All vaccines were tested with and without FIA, implying that the experiment included 8 groups of fish. Each group contained 100 individuals. To allow mixing of the groups two by two, fish in 4 groups were tagged by clipping the adipose fin under anaesthesia. Each of the tagged fish groups was combined with a non-tagged fish group into one tank. The setup thus included 4 tanks with 200 fish representing 2 groups in each tank (**Supplementary Table 1**). Vaccinated fish were fed with pelleted trout feed (BioMar A/S) at a ratio of 2% biomass per day.

At 50 days post-vaccination (dpv), 10 fish from each group were randomly selected and terminated for blood sampling, while the remaining 90 fish were anaesthetized by immersion in water with benzocaine (0.01%) and tagged by operculum clips. Together with adipose fin clips, the operculum clips (upper or lower and right or left side, respectively) allowed unique tagging of each of the 8 groups (**Supplementary Table 1**). Challenge was performed in 6 replicate tanks each containing 15 fish from each vaccine group corresponding to 120 fish in total per tank. In five tanks, challenge was performed under anesthesia by IP injection of 100 µl TB containing 5 × 10^4^ CFU of *A. salmonicida* homologous to the vaccine strain. Fish in the last tank were injected with sterile TB broth as a non-infected control. The course of morbidity was recorded for 19 days. Moribund fish were collected 3 times per day. Humane endpoints were defined as abnormal swimming behavior and/or loss of balance at which point affected fish were euthanized and recorded as mortality. Representative numbers of euthanized fish from all aquaria and groups were examined bacteriologically to confirm infection by *A. salmonicida* as the plausible cause of disease. After several days without the presence of clinical disease, the experiment was terminated at 19 days post-challenge (dpc) by euthanizing all remaining fish.

The negative control fish injected with TB broth were blood sampled for examination of antibody levels at this time, corresponding to 69 days post-vaccination (dpv). To determine the prevalence of subclinical infection among the survivors following challenge, 5 surviving fish of each group in each of the five tanks challenged with *A. salmonicida* were examined bacteriologically by swapping the head kidney and plating on blood agar plates.

### 2.8 Vaccination trial-2

Sixty rainbow trout (average weight 409g) were maintained at 12°C in two 500L tanks supplied with air and running tap water and water recirculation through an external mechanical/biological filter unit (Eheim). After anesthetizing the fish with benzocaine (0.01%), fish were intraperitoneally injected with 100µl RGNNV VLP-VapA-SpyCatcher (104µg), naked RGNNV VLP mixed with VapA-SpyCatcher (39µg + 65µg, respectively), AP205 VLP-VapA-SpyCatcher (78µg), naked AP205 VLP mixed with VapA-SpyCatcher (15µg + 63µg, respectively), formalin-killed *A. salmonicida* (2 × 10^6^ CFU) or buffer (Tris-HCl). For selected antigens, vaccination with 1:1 (v:v) emulsifications in FIA was also included. Each vaccine group included 6 fish individually tagged with passive integrated transponder (PIT) tags implanted into the epaxial muscle. Each tank included 3 fish from each vaccine group. The fish were fed with a 2% body weight daily ratio of pelleted feed (BioMarA/S).

At 48 days post-vaccination, the fish were terminated by immersion in 0.01% benzocaine and blood was sampled from the caudal vein. After incubation at room temperature for 1 h and at 4°C overnight, the samples were centrifuged at 5000 × g for 10 min, and the sera were collected and stored at -80°C until analyzed.

### 2.9 Preparation of whole cell antigen for ELISA

*A. salmonicida* strain 090710-1/23 was grown in 50ml of TB broth for 48 h at 20°C before the bacterial cells were collected by centrifugation at 3000 × g for 20 min at 4°C. The pellets were resuspended in 4ml of PBS and sonicated at an amplitude of 15 microns (corresponding to 2 x 10^13^ Hz) on ice for 10 cycles of 30 s on and 30 s off (Soniprep 150, MSE). The protein concentration was measured using the BCA kit.

### 2.10 Lipopolysaccharide antigen purification for ELISA

*A. salmonicida* strain 090710-1/23 was grown in 50ml of TB broth for 48 h at 20°C without shaking. The culture was centrifuged at 3000 × g for 20 min at 4°C, and the supernatant was discharged. The pellet was resuspended in 400µl of a solution of 0.5% SDS Laemmli buffer in PBS and vortexed until no bacterial aggregations were observed. The resuspended pellet was incubated at 90°C for 10 min, followed by incubation on ice for 5 min. Afterward, two sequential enzymatic treatments were carried out: first, with 5µl of DNase I (10mg/ml) incubated for 1 h at 37°C, and then with 10µl of Proteinase K (20mg/ml) incubated for 3 h at 60°C. After enzymatic treatments, 1ml of Triazol reagent (Invitrogen) was added and the sample was vortexed for 30 s. Subsequently, 200µl of chloroform was added, vortexed for 30 s, and incubated for 10 min at room temperature before centrifugation at 12000 × g for 10 min. After centrifugation, 400- 600µl of the aqueous phase, without including any precipitated DNA, were collected in a 2ml tube, mixed with 2 volumes of cold 0.375M of magnesium chloride in 95% ethanol, and incubated at -20°C for 1h. After incubation, the solution was centrifuged at 16000 × g for 10 min at 4°C. The supernatant was discharged. The pellet was washed with 500µl of 70% ethanol and centrifuged again at 16000 × g for 10 min at 4°C. The pellet was resuspended in 200µL of PBS and vortexed until no pellet was observed. Then, 500µl of Trizol were added, the sample vortexed for 30 s followed by adding 100µl of chloroform on topvortexing for 30s, and incubation for 10 min at room temperature. The sample was subsequently centrifuged at 12000 × g for 10 min, the aqueous phase collected, and the magnesium chloride precipitation and ethanol washing steps repeated. The resulting pellet was resuspended in 200µl PBS and stored at -20°C until analysis. This protocol was based on Yi, et al., 2000 (14).

### 2.11 Examination of the antibody response by ELISA

The wells in 96-well NUNC Maxisorb ELISA plates were coated overnight at 4°C with 50 μl of the sonicated *A. salmonicida* (5μg/ml), purified VapA-SpyCatcher (5μg/ml), purified LPS (not quantified directly, but corresponding approximately to the LPS in wells coated with *A. salmonicida* sonicate) all diluted in coating carbonate buffer 0.1M (pH 9.6), or with coating buffer without antigen. Following three times washing with PBS-T (0.05% tween20 in PBS), 100µl/well of PBS-T-skimmed milk (5% skimmed milk and 0.05%Tween20 in PBS) was added and the plates incubated for 1 h at room temperature. After three times rinsing, 50µl of fish sera, five times serial diluted in PBS-T-skimmed milk starting from a 1/100 dilution, were added to duplicate wells followed by incubation overnight at 4°C. After three times washing, monoclonal antibody 4C10 anti-trout IgM (15) hybridoma supernatant diluted 1/50 in PBS-T-BSA (1% BSA and 0.05% Tween20 in PBS) was added and plates incubated for 1 h at room temperature. Finally, after another three times washing, peroxidase-conjugated rabbit anti-mouse immunoglobulins (P0260, DAKO) diluted 1/1000 in PBS-T-BSA was applied to the wells and plates incubated for 1 h at room temperature. After three times washing, 50μl of peroxidase substrate, TMB plus (Kem-En-TecNordic A/S), was added and the plates were incubated at room temperature for 10- 15 min in the dark. The reaction was stopped by adding 50µl 1N sulfuric acid and absorbance was measured at 450nm and 650nm by ELISA reader (Synergy HT, Biotek) using the Bio Tek Gen5 software (Agilent). The well readout was calculated as absorbance at 450nm minus absorbance at 650nm. Sample readout was calculated as the average of two replicate wells, and for the 1/100 dilutions readout in wells without antigen was subtracted from the readout of wells coated with antigen to eliminate signal from non-specific antibody binding.

### 2.12 Statistics

Challenge experiment: Statistical analyses of the protection against bacterial challenge in vaccination Trial-1 the was performed in R (v. 4.2.0, http://www.R-project.org/). Logistic regression assuming a binomial distribution was used to compare the effect of the different vaccines on the probability of the fish to survive throughout at the experiment. The R packages used were “glmmTMB” (v. 1.1.4) (16) and “emmeans” (v. 1.8.2, https://CRAN.R-project.org/package=emmeans). The variability between tanks was considered as random effect. Odd ratios >1 with a p-value <0.05 were considered to reflect statistical difference between vaccine groups.

Antibody reactivity: assuming an non-normal distribution of the data due to limited number of observations and several outliers, the non-parametric Kruskal-Wallis test and the *post hoc* Dunn’s multiple comparisons test were applied using GraphPad Prism version 8.3.0 (USA) to examine the statistical significance of differences between groups in terms of antibody reactivity in ELISA. Comparisons giving p-values <0.05 were considered to reflect significant difference.

## 3 Results

### 3.1 Production of VLPs and VapA-SpyCatcher

**Figure 1:**
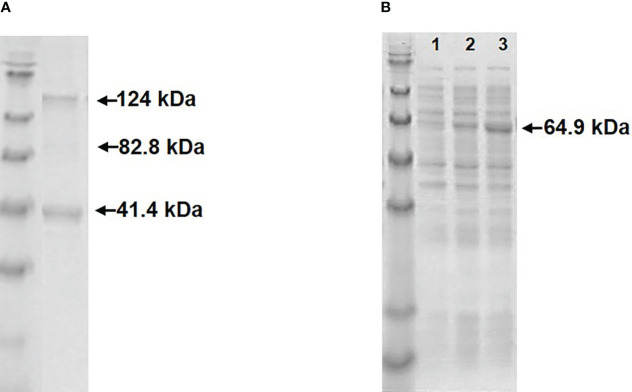
Visualization of recombinant proteins by SDS-PAGE. **(A)** Upon treatment with SDS-sample buffer purified RGNNV VLP-SpyTags dissolved into two major bands, corresponding to the Cp-SpyTag monomer (41.4 kDa) and trimer (124 kDa) along with a faint dimer band (82.8 kDa). **(B)** Supernatants from sonicated *E. coli* cells expressing VapA-SpyCatcher (64.9 kDa) at 37 °C (lane1), 27 °C (lane2), or 20 °C (lane3).

Expression of RGNNV Cp-SpyTag and VapA-SpyCatcher by transformed *E.coli* following induction with IPTG resulted in intracellularly accumulated recombinant proteins which could be released by cell disruption by sonication. The RGNNV Cp-SpyTag protein formed VLPs, which could be purified by ultracentrifugation. The subsequent SDS-PAGE analysis revealed two major bands corresponding to the Cp monomer (41.4 kDa) and trimer (124.2 kDa) (**Figure 1A**). The optimal temperature for the expression and subsequent assembly of the RGNNV Cp-SpyTag protein into VLPs was 20°C. Expression of soluble VapA-SpyCatcher protein (64.9 kDa) was similarly temperature-dependent. Although high levels of expression could be achieved at 37°C, a major part of the protein ended up in intracellular inclusion bodies at this temperature. The optimal temperature for achieving soluble VapA-SpyCatcher from sonicated cells was thus 20°C regardless of the IPTG concentration (**Figure 1B**). The expression of AP205 Cp-SpyTag and subsequent purification of VLPs was described earlier (7).

### 3.2 Development and characterization of VapA-decorated VLP vaccines

**Figure 2:**
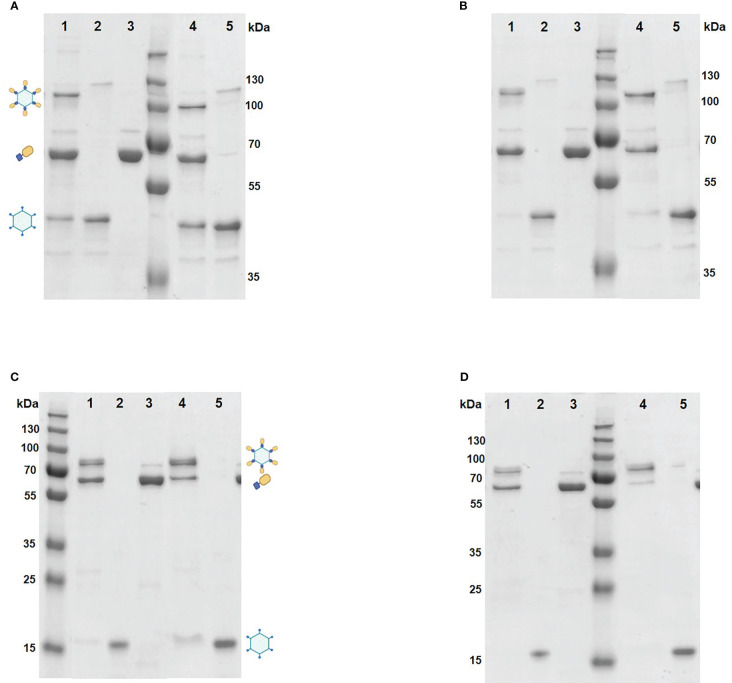
Coupling of VapA to VLPs analyzed by SDS-PAGE. The coupling reactions were performed by mixing purified RGNNV VLP-SpyTag (lane2 and 5) and VapA-SpyCatcher (lane3) at 4 °C for 2 h **(A)** or 24 h **(B)** in 1:1 (lane1) or 2:1 (lane 4) molar ratio. AP205 VLP-SpyTag (lane 2 and 5) and VapA-SpyCatcher (lane 3) were mixed in 1:1 (lane 1) or 2:1 (lane 4) molar ratio at 4°C for 2 h **(C)** or 24 h **(D)**. Following electrophoresis, gels were stained with Coomassie blue followed by densitometric analysis. The VLPs dissolved into Cps upon treatment with SDS-PAGE sample buffer. The covalent coupling of VapA-SpyCatcher (65 kDa) to the Cp-SpyTag resulted in formation of the VapA-Cp fusion protein and band shift of the Cp-SpyTag from 45 kDa to 110 kDa for the RGNNV Cp **(A, B)** and from 15 kDa to 80 kDa for the AP205 Cp **(C, D)**. Symbols indicate bands corresponding to the Cp-SpyTag, the VapA-SpyCatcher, and the Cp(SpyTag)-VapA(SpyCatcher) fusion proteins.

**Figure 3:**
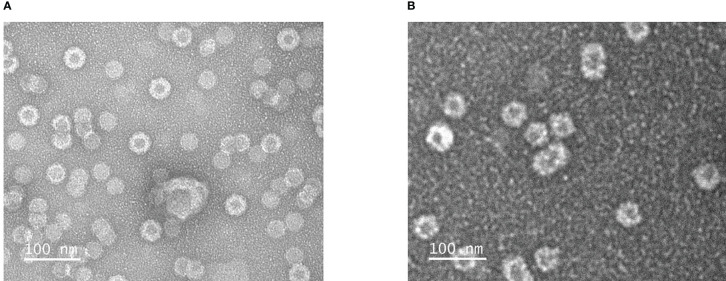
Transmission electron microscopical images of negatively stained RGNNV VLPs **(A)** and RGNNV VLPs decorated VapA-SpyCatcher **(B)**.

**Figure 4:**
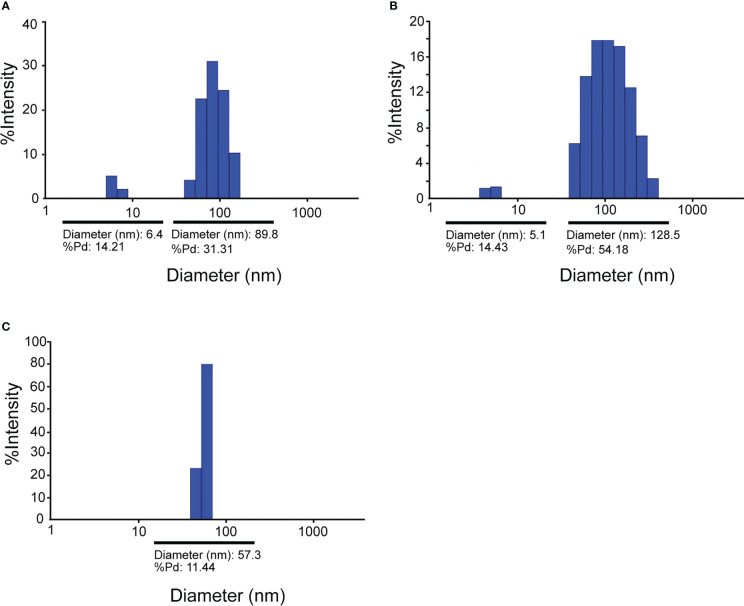
Dynamic Light Scattering (DLS) analysis of RGNNV VLPs **(A)**, RGNNV VLPs decorated with VapA-SpyCatcher **(B)**, and AP205 VLPs decorated with VapA-SpyCatcher **(C)**.

Mixing of purified VapA-SpyCatcher with VLPs displaying SpyTags on their surface resulted in covalently linking of VapA-SpyCatcher and Cp-SpyTag proteins. The coupling reactions resulted in proteins of the combined size of VapA-SpyCatcher (69.1 kDa) and RGNNV Cp-SpyTag (41.4 kDa) or VapA-SpyCatcher and AP205 Cp-SpyTag (16.5 kDa), corresponding to fusion proteins of 110.5 kDa and 85.6 kDa respectively, as visualized by SDS-PAGE (**Figure 2**). The samples were subjected to a stability spin test (16000 × g, 2 min), showing no loss of the coupling band, indicating that the vaccines were stable and not prone to precipitation nor to aggregation (data not shown). A major fraction of the Cp-SpyTag proteins appeared to become engaged with VapA-SpyCatcher as visualized by an upwards shift of the Cp-SpyTag bands. Densitometric gel scans accordingly indicated a 95% coupling efficiency at 1:1 Cp-SpyTag : VapA-SpyCatcher molar mixing ratio after 24 hours coupling for the RGNNV Cp-SpyTag (**Figure 2B**) and a 99% coupling efficiency for the AP205 Cp-SpyTag under the same conditions (**Figure 2D**). Coupling efficiency was almost similar with a 2:1 molar ratio with some uncoupled VapA-SpyCatcher still remaining. This suggested that the VapA-SpyCatcher protein concentration had been underestimated. The non-combined VapA-SpyCatcher was removed either by dialysis or by ultracentrifugation and elimination of excessive VapA-SpyCatcher was confirmed by SDS-PAGE (**Supplementary Figure 1**). Examining the RGNNV VLP shape and size by TEM confirmed the presence of virus-like particles of approximately 30 nm in diameter before and 40 nm after coupling, respectively (**Figure 3**). Size homogeneity analysis by DLS showed that the RGNNV VLPs had a higher degree of polydispersity (Pd% ~50) compared to the more monodispersed AP205 VLPs (Pd% ~12). For both VLPs the DLS analyses revealed an increased diameter following VapA decoration, increasing from 36 nm to 57 nm and from an average of 90 nm to 130 nm for AP205 and RGNNV, respectively. The rather large size difference of the DLS estimated diameters of the two VLPs despite the similar sizes based on EM examinations probably reflects that DLS size estimates depend on particle surface structures and the degree of sample polydispersity (**Figure 4**).

To examine whether freezing and thawing affected the stability of the decorated VLP vaccine, the effect of repeated freezing and thawing up to 3 times was analyzed (**Supplementary Figure 2**). Based on densitometry scans of SDS-PAGE gels the first cycle resulted in a quantitative loss of 20% possibly due to hard to dissolve aggregates. However, the integrity of the conjugates appeared stable with unchanged relative amounts of CP-SpyTag and VapA-SpyCatcher bands throughout the 3 cycles.

### 3.3 Protection against challenge with *A. salmonicida*

**Figure 5:**
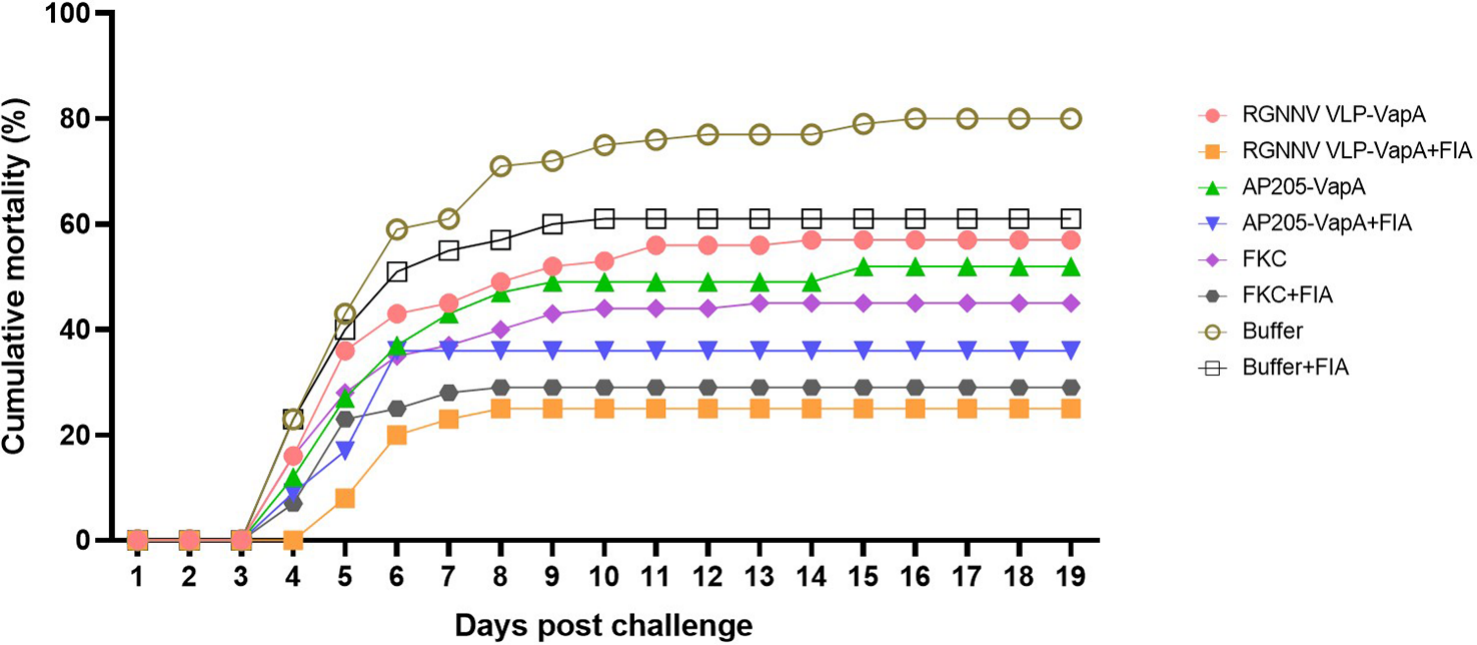
Cumulative mortality after challenge of vaccinated rainbow trout with *A. salmonicida*. The fish were vaccinated with RGNNV VLP-VapA, AP205 VLP-VapA, FKC, or buffer, with or without Freunds incomplete adjuvant (FIA). At 50 dpv, fish were challenged by IP injection of 5×10^4^ CFU of *A. salmonicida*. After challenge, diseased/moribound fish were continuously collected and registered on a daily basis. Significances of differences between survival of the groups are specified in **Table 2**.

In vaccination Trial 1, the fish were exposed to *A. salmonicida* by IP injection. Clinical disease demanding termination of the affected individuals started 4 dpc. At 16 dpc no further diseased fish occurred and the experiment was terminated at 19 dpc. Mortality reached 80% in the buffer-injected control group on average. Results are shown in **Table 2** and **Figure 5**. Variability between the replicate tanks was relatively high but with 5 replicate tanks robust statistics could still be performed (**Table 2**). On average, trout immunized with FIA-formulated RGNNV VLP-VapA displayed a relative percentage of survival (RPS) value of 69% followed by groups given FIA-formulated FKC (64%) and FIA-formulated AP205 VLP-VapA (55%) (**Table 3**). While these 3 groups were not significantly different from each other, they were all significantly different from the group given FIA-formulated buffer (average RPS=24%). The highest RPS value among the groups given vaccines without FIA was 44%, reached by the FKC. This was followed by the group receiving AP205 VLP-VapA (35%) and the group getting RGNNV VLP-VapA (29%) (**Table 3**). While these were not significantly different from each other, they were all significantly different from the buffer control (**Table 2**). Bacteriological examination of representative numbers of clinically diseases fish in all vaccine groups resulted in re-isolation of *A. salmonicida*, whereas no bacterial infection was detected in the healthy survivors terminated at the end of the trial.

**Table 2:** Statistical analyses of significances of differences in survival between the vaccine groups after challenge of the fish with *A. salmonicida* in vaccination Trial 1.

Group comparisons Group vs Group		Odd ratio	*p*. value
Buffer+ FIA	RGNNV VLP-VapA+FIA	5.604	**<.0001**
Buffer+ FIA	AP205 VLP-VapA+FIA	3.223	**0.0047**
Buffer+ FIA	FKC+FIA	4.475	**0.0002**
RGNNV VLP-VapA+FIA	AP205 VLP-VapA+FIA	0.575	0.4228
RGNNV VLP-VapA+FIA	FKC+FIA	0.799	0.9298
AP205 VLP-VapA+FIA	FKC+FIA	1.388	0.7903
Buffer	RGNNV VLP-VapA	2.62	**0.0467**
Buffer	AP205 VLP-VapA	3.56	**0.0031**
Buffer	FKC	4.63	**0.0002**
RGNNV VLP-VapA	AP205 VLP-VapA	1.36	0.7887
RGNNV VLP-VapA	FKC	1.77	0.3130
AP205 VLP-VapA	FKC	1.30	0.8492

Odd ratios >1 with a p-value <0.05 were considered statistically different.

Bold numbers indicate significant protection compared to buffer control (with or without FIA).

**Table 3:** Survival rates of vaccinated fish following challenge with *A. salmonicida* in vaccination Trial 1.

Vaccine group	Rep.1	Rep.2	Rep.3	Rep.4	Rep.5	Average	RPS*
RGNNV VLP-VapA	40%	47%	33%	50%	36%	43%	29
RGNNV VLP-VapA+FIA	67%	60%	93%	81%	75%	75%	69
AP205 VLP-VapA	40%	33%	73%	47%	50%	48%	35
AP205 VLP-VapA+FIA	60%	47%	87%	93%	33%	64%	55
FKC	47%	69%	47%	47%	67%	55%	44
FKC +FIA	53%	67%	69%	80%	87%	71%	64
Buffer	0%	47%	33%	13%	13%	20%	0
Buffer+FIA	29%	60%	33%	47%	20%	39%	24

*Relative percentage of survival = 100 x (1-(mortality in vaccine group/mortality in buffer control group)).

Challenge was performed in 5 replicate tanks, each including 15 individuals from each vaccine group.

### 3.4 Antibody response

**Figure 6:**
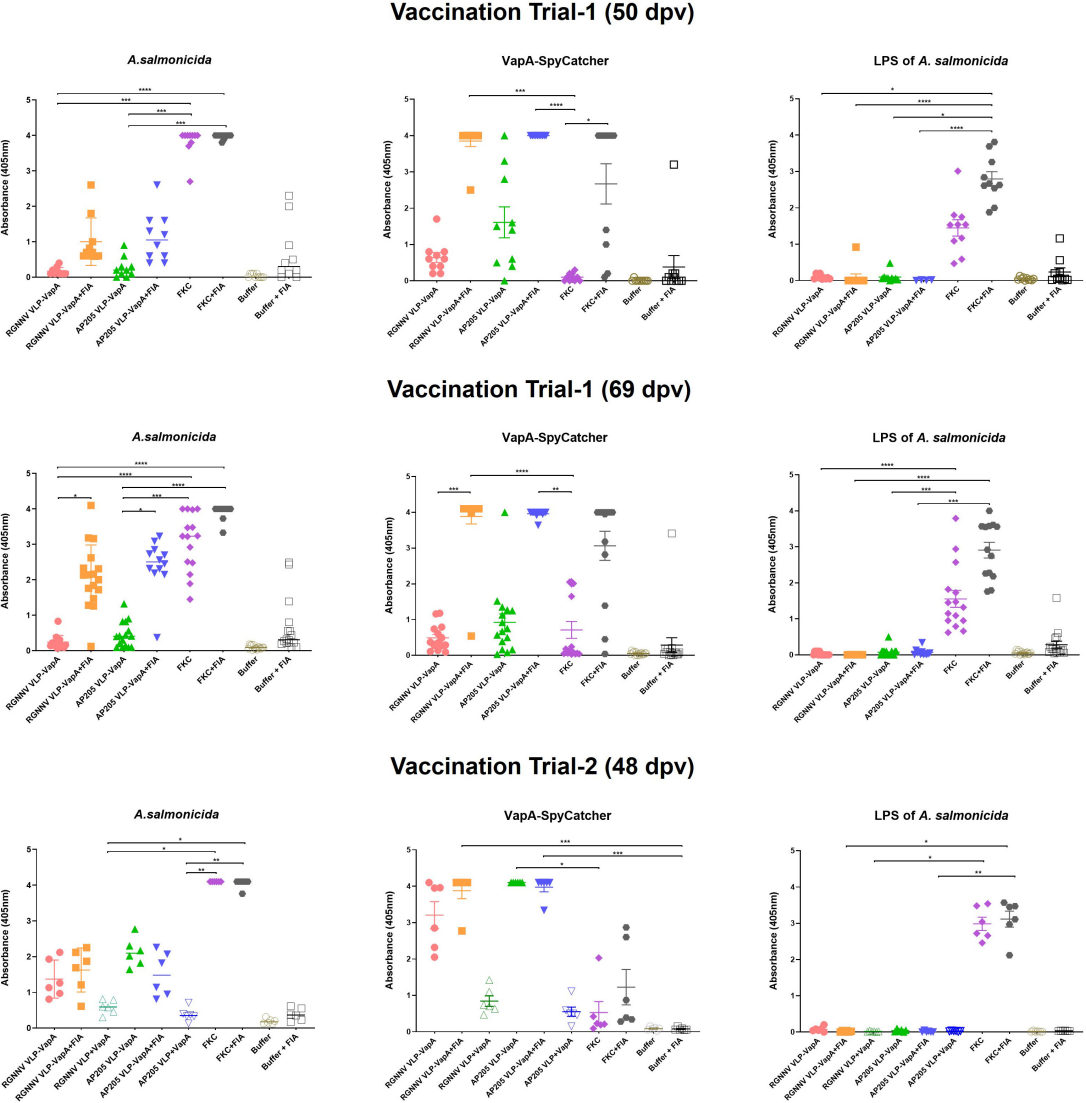
Antibody reactivities in sera from vaccinated rainbow trout. Sera were diluted 1/100 and examined by ELISA for reactivity with sonicated *A. salmonicida* cells, purified VapA-SpyCatcher, and purified *A. salmonicida* LPS. In vaccination Trial 1, fish were vaccinated with RGNNV VLP-VapA, AP205 VLP-VapA, FKC, or buffer with or without FIA, and sera were sampled 50 and 69 dpv. The vaccination Trial 2 sera were sampled 48 dpv. In addition to the vaccines in Trial 1, Trial 2 included two groups of fish given RGNNV VLP+VapA-SpyCatcher (without coupling) or AP205 VLP+VapA-SpyCatcher (without coupling), respectively. Significance of differences between selected groups are indicated by asteriscs *p ≤ 0.05; **p ≤ 0.01; ***p ≤ 0.001). Significance of differences between all groups are specified in **Supplementary Tables 2-4**.

**Figure 7:**
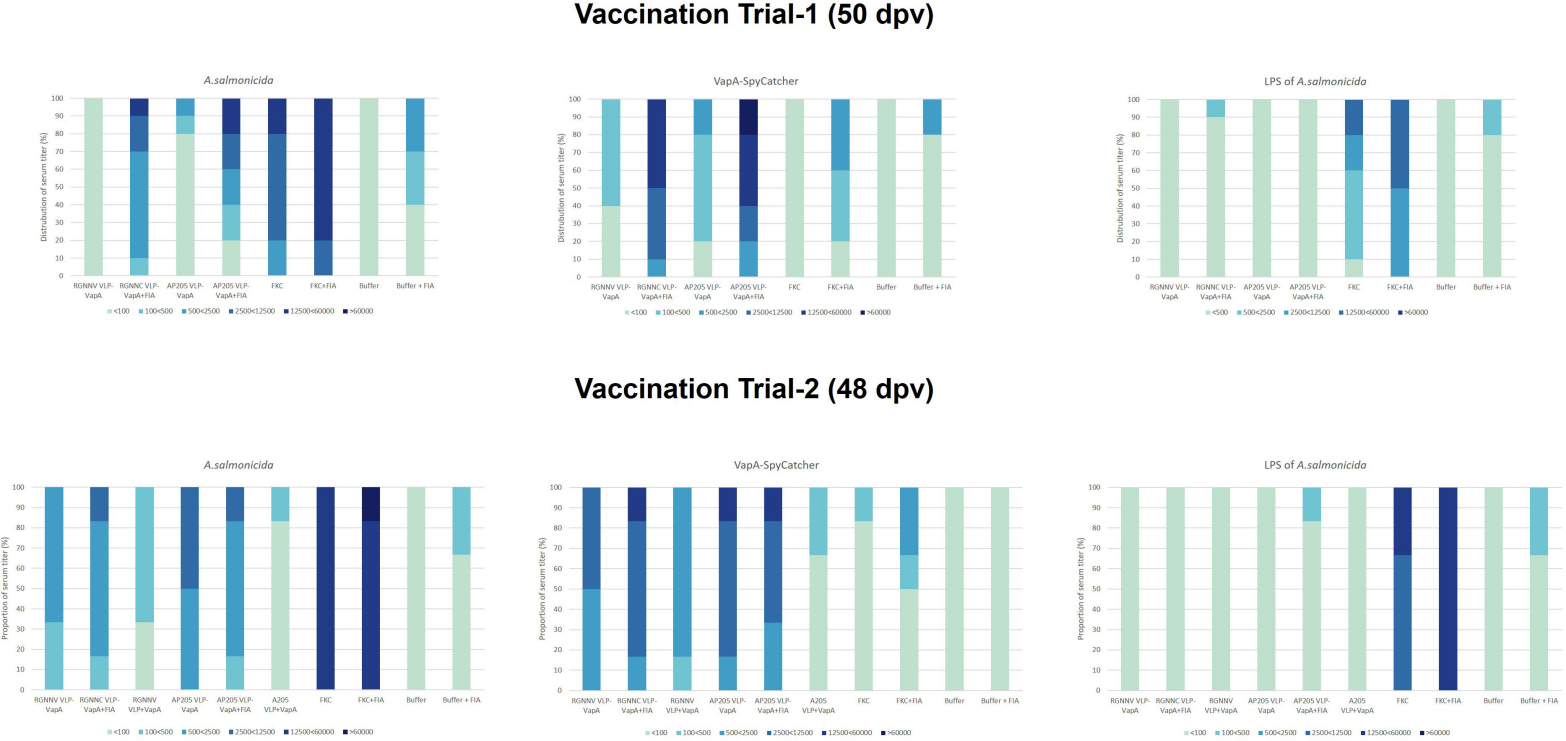
ELISA antibody titer distributions in groups of fish sampled in vaccination Trial 1 (n=10, 50 dpv) and vaccination Trial 2 (n=6, 48 dpv). Sera were examined in 5-fold dilutions, starting with 1/100 and ending by 1/60000. Antigens included sonicated *A. salmonicida* cells, purified VapA-SpyCatcher, and purified *A. salmonicida* LPS.

In the vaccination Trial 2 where large rainbow trout (average weight 409g) were injected with VLPs mixed with uncoupled VapA-SpyCatcher or VLPs decorated with VapA by the SpyTag/SpyCatcher conjugation, the results demonstrated that linking to the VLPs was essential for immunogenicity of VapA-SpyCatcher. Only low levels of VapA-SpyCatcher reactive antibodies could thus be detected even in the lowest tested serum dilution (1/100) in fish injected with VLPs without SpyTag mixed with VapA-SpyCatcher. In contrast, high VapA-SpyCatcher specific antibody reactivities were seen in sera from fish injected with VapA-decorated VLPs (**Figures 6**, **7**) (**Supplementary Tables 2**–**4**). Interestingly, fish given the AP205 VLPs decorated with VapA had higher antibody reactivities and titers than fish injected with VapA-decorated RGNNV VLPs (**Figures 6**, **7**). And whereas emulsification with FIA resulted in a stronger antibody response for the latter, this was not the case for the AP205 VLPs. Although not the same, a similar trend was observed in the antibody response profiles among the small rainbow trout (13 ± 2g) used in the vaccination Trial 1. Here, inclusion of FIA was essential for induction of a strong antibody reactivity against VapA-SpyCatcher by VapA-decorated RGNNV VLPs with only 1 out of 10 examined fish in the group injected with VLP-VapA without adjuvant giving a signal above 1.0 in ELISA compared to 6 out of the 10 tested fish injected with AP205 VLP-VapA at 50 dpv (**Figure 6**). In the vaccination Trial 1, emulsification of AP205 VLP-VapA with FIA had a more radical effect than seen in the vaccination Trial 2, leading to a strong antibody response to VapA-SpyCatcher in all examined individuals (**Figures 6**, **7**). While strong reaction with VapA-SpyCatcher generally was reflected by intermediate reactivity with sonicated *A. salmonicida*, VLP-VapA vaccinated fish displayed no or very low antibody reactivity with LPS. In contrast to this, most fish injected with FKC responded to *A. salmonicida* and LPS in ELISA, while only fish receiving FKC emulsified in FIA mounted a detectable antibody response also to VapA. And among these, some individuals (in both trials) did not have VapA binding antibodies even in the lowest tested dilution (1/100) (**Figures 6**, **7**).

In vaccination Trial 1, serum samples were also collected from non-challenged controls at the end of the challenge trial, 69 days post-vaccination. At this time, antibody reactivities tended to be higher compared to 50 dpv, particularly in the groups given vaccines formulated with FIA. Accordingly, a higher ratio of both VLP-VapA vaccinated groups now displayed a stronger signal with the *A. salminocida* antigen, while the FKC vaccinated group had more fish reacting with the VapA antigen (**Figure 6**). A few outliers displaying antibody reactivity above 1.0 against *A. salmonicida* and VapA-SpyCatcher in the group vaccinated with Buffer+FIA was likely due to incorrect interpretation of the tag clippings in this group. No similar pattern was seen in the corresponding group in Trial 2 (**Figure 6**).

## 4 Discussion

We here report high immunogenicity of VLPs decorated with the *A. salmonicida* VapA protein in rainbow trout. While VLPs are well known as potent vaccine candidates against diseases caused by the parental viruses in both mammals and fish (1, 2), studies on the use of VLPs as generic antigen delivery vehicles in fish are limited to preliminary reports (5, 8). A covalent conjugation system based on an intramolecular iso-peptide bond found in a *Streptococcus pyrogenes* adhesion protein, and named SpyTag and SpyCatcher refering to the smaller and larger peptide fragments respectively, was earlier developed by Zakeri et al. (2010) (17, 18). The SpyTag/SpyCatcher conjugation system was later applied for decorating VLPs with malaria antigens or autologous cancer antigens and shown to induce a strong antibody response in mice even after a single injection (6, 7). Head to head comparison of two different VLPs, namely human papillomavirus and *Acinetobacter* phage 205 VLPs indicated superiority of the latter in terms of induction of specific IgG antibodies in mice (19). This led to development of one of the recent vaccine candidates against COVID-19 based on AP205 VLPs decorated with the receptor binding domain of the SARS CoV-2 virus´ spike protein (20). For vaccination of farmed fish, a single injection should preferably provide life-long immunity taking the production cycle into account. Based on the promising results in mice (6, 7, 19) we wanted to explore this vaccine concept for induction of immunity in rainbow trout, representing one of the most important salmonid fish species in aquaculture. The *Aeromonas salmonicida* VapA protein was used as model antigen. *A. salmonicida* sub-species *salmonicida* is known as the causal agent of furunculosis, a septicemic bacterial disease in salmonids, and although traditional bacterin-based vaccines for Atlantic salmon are available and may be used for protecting rainbow trout against the disease, the protection is incomplete and associated with side effects (10, 21). The *A. salmonicida* bacterial cell is characterized by having an outer layer composed of the virulence array protein A (VapA) and LPS (11, 22). Both contribute to the induction of specific antibodies correlating with protective immunity (23, 24), and partial protection following vaccination with purified VapA as well as with a recombinant adenovirus encoding VapA has been reported (24–26).

### 4.1 The antigen-decorated VLP vaccine platform

Apart from allowing tailored linking of larger antigens to the VLP surface, one advantage of the SpyTag/SpyCatcher concept is that the viral Cp and the antigens can be expressed separately according to individually optimized conditions and, if needed, in different expression platforms. We here used *E. coli* for expression of both Cps and VapA, but it was necessary to individually optimize the expression conditions for optimal yield. Furthermore, the self-assembly of the Cps into VLPs makes their purification by differential centrifugation, size separation or other simple techniques cost-efficient and generic due to their particulate nature. The high and specific affinity of the SpyTag/SpyCatcher peptides subsequently allows specific conjugation of a selected antigen to the surface of the VLPs, which subsequently can be purified as antigen-decorated VLPs. We here compared VLPs based on the bacteriophage AP205 and the fish betanodavirus RGNNV capsid proteins. As mentioned above, the AP205 VLPs have earlier been shown to be superior to VLPs based on the human papillomavirus 16 L1 capsid proteins in terms of triggering a specific antibody response to the antigen conjugated to the VLP surface (19), whereas the antigen-decorated RGNNV VLPs were only described recently (8). Like the AP205 VLPs, RGNNV VLPs consist of 180 Cp molecules arranged triangular subunits (T=3) and are 30-35 nm in diameter, without antigen decoration (27). Based on densitometry scan of SDS-PAGE gels, both VLPs showed high coupling efficiency with 95% of the Cp molecules being conjugated to VapA for the RGNNV VLPs and 99% for the AP205 VLPs. This difference was also reflected in the dynamic light scattering (DLS) analysis showing a higher heterogeneity or polydispersity in the RGNNV VLP preparations both before and after VapA conjugation, whereas AP205 VLP preparations appeared homogenous or monodispersed with and without VapA (**Figure 4**). Nodavirus CPs have been described to trimerize in the initial VLP-assembly processes (27), whereas the AP205 CPs initially tend to engage in dimers (28). Whether these differences might affect the efficiency of the VLP auto-assembly procedure of the Cp-SpyTags fusion proteins or influence structural features like the number of accessible SpyTag peptides on the VLP surface remains to be examined. However, as earlier shown for the AP205 VLPs (12) electron-microscopical examination of the RGNNV VLPs revealed particles of the expected size and shape both before and after VapA conjugation (**Figures 3**, **4**).

### 4.2 The antibody response

The presentation of VapA on the surface of VLPs promoted a strong and specific antibody response in the immunized/vaccinated fish compared to very low antibody levels in sera from fish given the same amounts of VLP mixed with VapA in an un-conjugated form. This is in accordance with earlier studies in mice immunized with malaria antigens and demonstrated that also in fish, immunogenicity in terms of triggering a strong antibody response is promoted by repetitive antigen presentation on VLPs (29). As known for traditional bacterin-based fish vaccines (30), we saw a significantly stronger and more long-lasting antibody response to VapA-decorated VLPs when these were emulsified in oil adjuvant. This could be due to an inflammation stimulating and/or depo-effect of the oil and further studies are needed to determine whether adjuvants causing less side effects could similarly support the immune response to the antigen-decorated VLPs. A similar effect was seen with the Squalene-Water-Emulsion (AddavaxTM) adjuvant in mice immunized with AP205 VLPs decorated with *Plasmodium* antigen (6).

Despite the administration of quantitatively equal amounts of VapA, a somewhat higher immunogenicity of the VapA-decorated AP205 VLPs compared to RGNNV VLPs was observed when fish were vaccinated without FIA. One explanation for this difference could be the above-mentioned qualitative differences reflecting a denser antigen coating along with a higher fraction of homogenously coated AP205 VLPs. Although long-term kinetics of the antibody response was not included in our study, the higher antibody titers in serum sampled 69 days post-vaccination from fish given FIA-formulated AP205 VLP-VapA indicated a high potency of this vaccine also for induction of long-lasting immunity.

While the VLPs are not packed with viral genomes, the auto-assembly in the Cp-expressing in *E. coli* is known to result in the packing of cytoplasmic bacterial elements like RNA, which are expected to provide innate immune stimulation along with potential adjuvant effect in mice (31). Whether this is also the case in fish remains to be examined.

The differences between antibody responses induced by the VapA-VLPs in larger (400g) and smaller (13g) fish might be explained by differences in vaccine/adjuvant doses per g body weight rather than by differences in immune competence since the response tended to be stronger in the small fish. Similar dose/size-related differences have been reported for DNA vaccines against viral diseases (32), and dose-response and time-course experiments will be needed to determine the optimal dose of VLPs decorated with antigens. The selected VLP/antigen doses used here were based on earlier RGNNV VLP vaccination trials in sea bass indicating 20-40µg VLP as the optimal dose (4) In this study, we examined antibody reactions and protection 48-50 dpv. Salmonid fish are expected to mount an adaptive immune response to vaccination within 400 degree-days (Temperature in Celsius x days) post-vaccination. Our fish were kept at 12°C and by having 48-50 days, corresponding to 576-600 degree-days between vaccination and challenge, we assumed to have allowed the fish time to raise an adaptive immune response.

### 4.3 Protective effect of antigen-decorated VLPs

Despite some inter-tank variability, possibly reflecting that a setup with more individuals per group in each tank would have been superior, both FIA-formulated VLP-VapA vaccines provided protection comparable to that observed with the FKC vaccine and significantly higher than the non-specific protective effect of the FIA emulsion alone (**Figure 5**; **Table 2**). The innate protective effect of FIA/oil adjuvant alone has been reported in earlier studies and may be due to upregulation of general antibacterial defense mechanisms like attracting/activating phagocytic cells (33). However, although the specificity of the protection in terms of challenge with a heterologous bacterial pathogen was not examined, the correlation with the induced antibody response suggests that vaccine-induced antibodies to VapA have contributed to protection. Accordingly, the AP205 VLP-VapA, which induced a significant antibody response in a major fraction of the fish even when administered without FIA, also induced significant protection when compared to control fish injected with buffer (**Figure 5**; **Table 2**). Partial protection with VapA purified from *A. salmonicida* cells has been reported earlier and our results thus confirm that VapA represents a good candidate for a recombinant vaccine against furunculosis in rainbow trout.

### 4.4 Limitations and prospects

Whereas the FKC vaccine also induced significant protection along with antibodies to VapA in most of the fish, our analyses of antibody response specificity suggested that LPS represented a dominant antigen in this vaccine, potentially overruling the response to VapA. Whether the more homogenous antibody response to VapA in the VLP-VapA vaccinated fish might result in more robust protection will have to be addressed by extended trials including more natural challenge routes such as co-habitation of vaccinated fish with *A. salmonicida* infected donors. Similarly, dose response trials with and without adjuvant in different sizes of fish along with trials testing duration of immunity are needed to determine the optimal use of the VLP-VapA vaccines. We here used purified VapA fused to SpyCatcher as antigen in ELISA for measuring the antibody response to VapA. Further studies with non-fused VapA will be required to examine whether the observed reactions could be partly due to antibodies recognizing the SpyCatcher component.

While VapA is the major structural protein in the outer A-layer of virulent *A. salmonicida*, other virulence components, including extracellular secreted proteases, might be relevant to include in the vaccine to obtain a further improved protection. Partial protection following vaccination has thus been reported for other recombinant *A. salmonicida* antigens or mixtures of such (34). The VLP SpyTag platforms easily allow combination of more antigens from one pathogen as well as formulation of polyvalent vaccines e.g. by mixing VLPs after antigen decoration. Follow up studies are planned to address these issues.

### 4.5 Final remarks

In conclusion, the results reported here demonstrate that antigen-decorated VLPs using the SpyTag/SpyCatcher technology for conjugation represent a promising approach for the generation of highly immunogenic fish vaccines based on recombinant protein antigens. And further that the VapA protein represents a potent candidate for such a vaccine against furunculosis in rainbow trout.

## Data availability statement

The original contributions presented in the study are publicly available. This data can be found here: https://figshare.com/s/43c5c101a67280b09f60.

## Ethics statement

The animal experiments were approved by the Danish Animal Experiments Inspectorate, Danish Ministry of Environment and Food, under the license No 2019-15-0201-00159.

## Author contributions

JIY, NL planned the study, acquired the funding and drafted the MS. JIY did the molecular clonings, recombinant VapA expression and VLP decoration, JIY and LG prepared the VLPs. JIY, DS, JS, IV and NL did the animal experiments, IV did the immunoassays, LG and AFS supervised the VLP work, all authors participated in the data analysis and revised the MS. All authors contributed to the article and approved the submitted version.
