# Supplementary material for: High immunogenicity of virus-like particles (VLPs) decorated with Aeromonas salmonicida VapA antigen in rainbow trout
Source: Front Immunol. 2023 May 22;14:1139206. doi: 10.3389/fimmu.2023.1139206 (PMC10239931; doi:10.3389/fimmu.2023.1139206)
Supplement: Supplementary file 1 [file DataSheet_1.docx]

High immunogenicity of virus-like particles (VLPs) decorated with *Aeromonas salmonicida* VapA antigen in rainbow trout

Jeong In Yang^1^, Dagoberto Sepúlveda^1^, Irina Vardia^1^, Jakob Skov^1^, Louise Goksøyr^2,3^, Adam Sander^2,3^ and Niels Lorenzen^1^*

^1^ National Institute of Aquatic Resources (DTU AQUA), Technical University of Denmark,2800 Lyngby, Denmark

^2^ Centre for Medical parasitology, Department of Immunology and Microbiology, University of Copenhagen, Copenhagen, Denmark

^3^ AdaptVac Aps, Copenhagen, Denmark

*** Correspondence:** Niels Lorenzen: nilo@aqua.dtu.dk

# Supplementary Figures


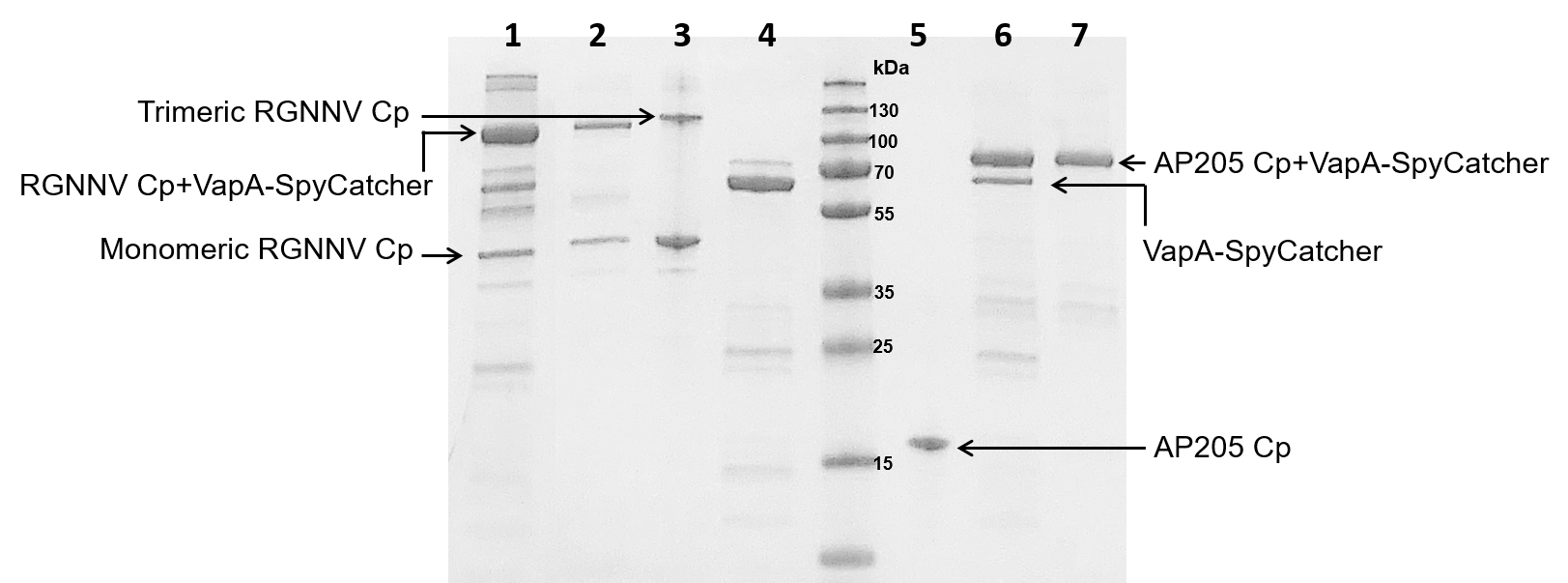


**Supplementary Figure 1.** Purification of VLPs decorated with VapA-SpyCatcher by ultracentrifugation. To remove excessive VapA-from coupling mixtures with either RGNNV VLP or AP205 VLP, samples were ultracentrifuged after coupling followed by discharge of supernatants and resuspension of pellets. Coupling mixture of RGNNV VLP-SpyTag and VapASpyCatcher, before and after centrifugation (lanes 1 and 2), RGNNV-VLP (lane3), VapA-SpyCatcher (lane4), AP205 VLP (lane5), AP205 VLP-VapA SpyCatcher mixture (lane6), and AP205 VLP-VapA SpyCatcher after ultracentrifugation (lane7).


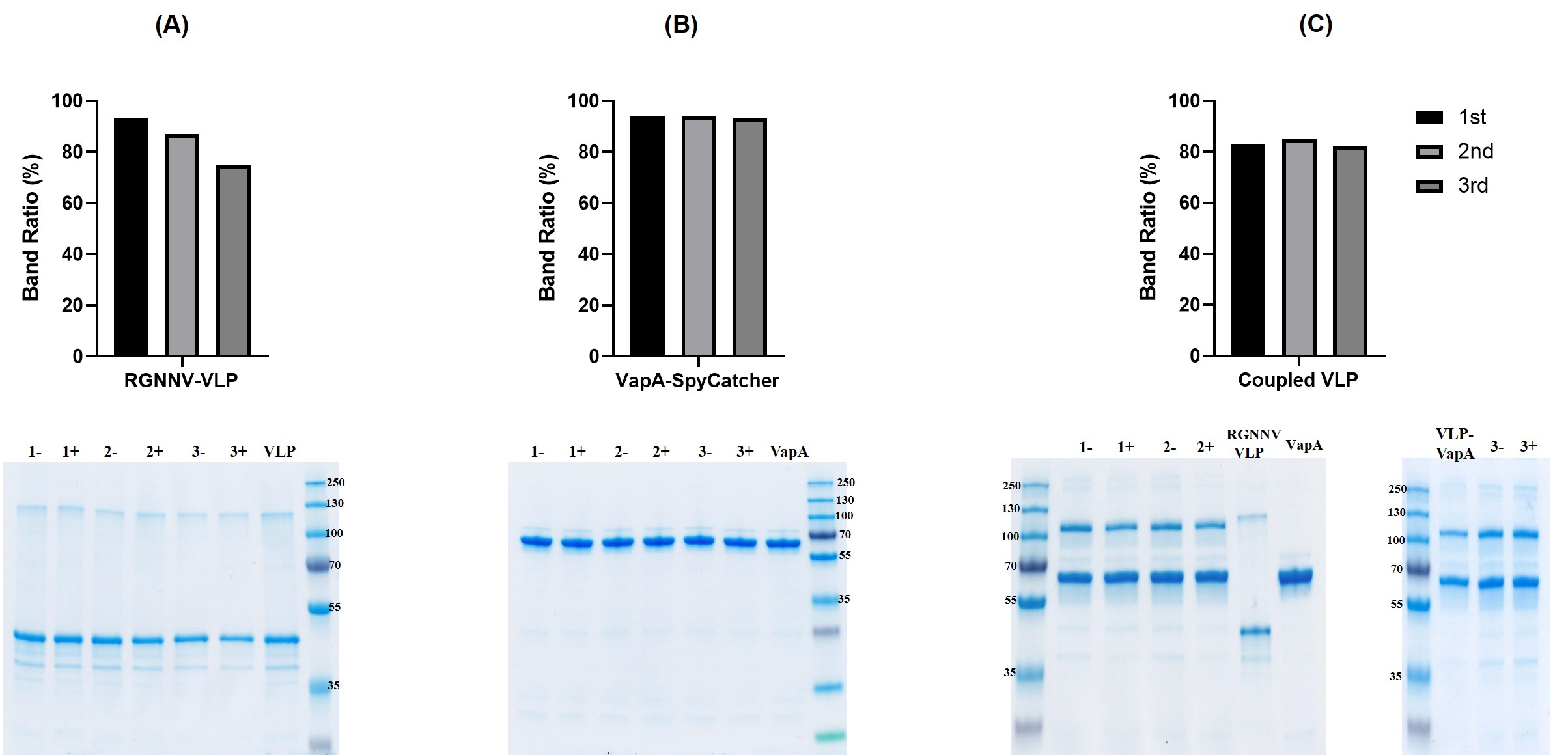


**Supplementary Figure 2.** Freezing and thawing stability test of RGNNV-VLP, VapA-SpyCatcher, and a mixture of the two following incubation for coupling.. The freezing and thawing cycle was performed three times (1st, 2nd, and 3rd), and samples were collected after each thawing. Aliquots were centrifuged and supernatants were compared with non-centrifuged samples in SDS-PAGE to examine for decreasing protein band intensity reflecting aggregation. . The bar diagrams show results of densitometric analysis of gel lanes loaded with centrifuged samples. The + or – on top of each lane refer to centrifugation or not, respectively.


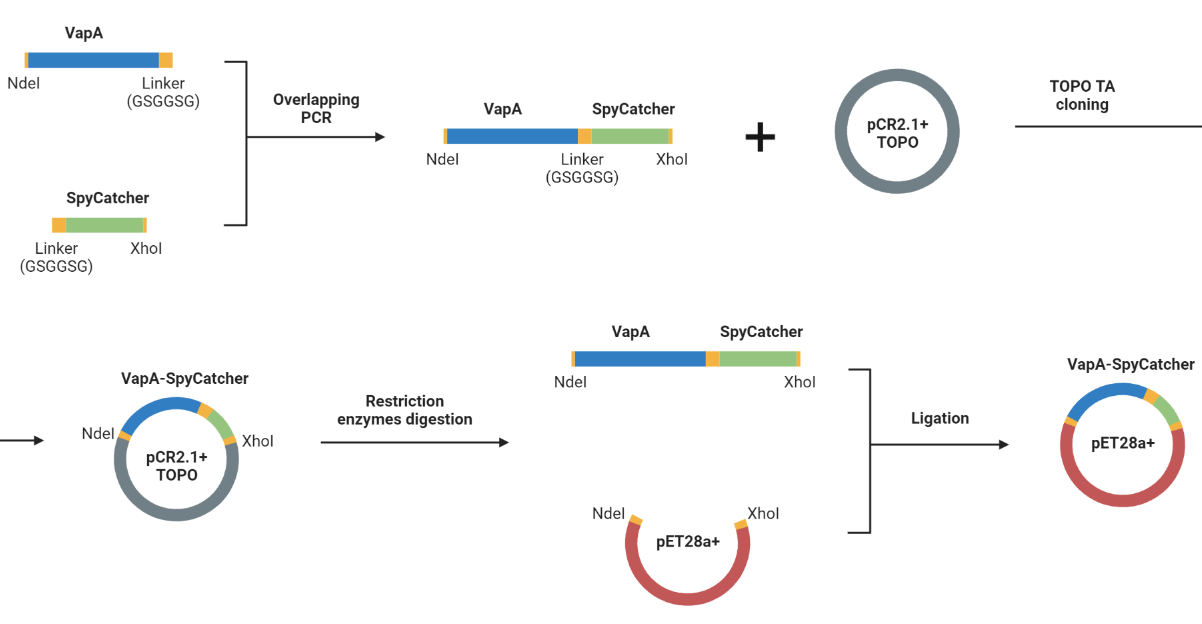


**Supplementary Figure 3.** VapA-SpyCatcher vector construction created with BioRender.com. The PCR-amplified VapA and SpyCatcher genes were connected by overlapping PCR using VapA_NdeI_F and SpyCatcher_XhoI_R primers (Table 1) and inserted into the pCR2.1+TOPO vector (Invitrogen). The VapA-SpyCatcher cassette was subsequently transferred into pET28a+ using the terminal NdeI and XhoI restriction sites. The VapA-SpyCatcher gene sequence is included below.

ATGTTTAAGAAGACTTTGATTGCAGCTGCCATTGTGGTCGGTTCCGCTGCACCTGCGTTTGCTGATGTCGTGATTAGCCCGAACGACAACACTTTCGTTACTACCTCCCTCGCATCTGTAACTAAGCAGCCGGTACTGGACTTCTCCACTGCTCAACAAAACCTGACCCTTAACTTCAGTGAAGTTGGTGACCTTAAGAACAACGGTTTCATTGTGTTGGAAATCCAAGGTGAAGGCCAATTCAACGACGCGGAAATCCGTCAGTGGCTGTCCAACGGTTTCTGGCGTAGGCCGTTTACCGGTCTGCTGGTTAACCCGAATGATCATGGTAATTTTGCCAATAGCGGTGAAGTTAATGACGTTCGGAAGTTCTTTAAGATTATTTCCGACGGTACCCAGCTGACCATCGTTCACACTATCGACAGCAATGGCAAGCGTCTGCGTCTTGCTCTTGCTTCTGATGTAGAAGAGACAATCAATTTTGCTGATGCAGAGGTTGAGCTGAAGCTGAACTTAGCTAACCAAGCCTTTAAGCTGACCTCCGGTTCTCAAGGTACAGTAGCTCTGACCGCAGGAGCTCTGTGGAACGCTTCTTACACTGCTGATCCGGTTGCTACCAAGCCGCTGTTCAAACTGGGTAAGCTGTTCCAGTTGAGTTTGACTAACGCTGGTAAAGCTACCGCTCTGGTTTCCGAAGGTTTCTTGAAACTTAATATCGGTGATGCGAATATTTCTGCTACTGATTTCGCGATTACCAACGTTACTACTAACCAGACCATCCAACGTGACAAGGTTAACCTGACCCTGACTGGTGATGTTTCTGCCTTCAAGAAAGATGCCAACGGTAACTTGGTAAACAAAGCTGGTGCTAGCATCGGTTGGAAAGCTGCTGCTGATGGTCAATCTGCTACAGCTGTCTTGGGTGCTGGCAACATGGCCGGTGGGGTTCAAAATGCTCTGGCTGCTTTTGGTACACTGTACGTTGCTGCAGATAACACTGTTCCGGTTCCTGCTGTTAACTTCAATGTTAAGGCTGAAATCCAAGGTGATAGCCAAGCTACCTATAACTACTTCAAGGACGAGCTGGCTGATCTCTTCATCCTCACCCGTGATGGTATGAAGTTTGACACAATTACTACTGGTACCACTTCTGCCAACCTCATCCACATTCGTGATGTATCTAACATCCTGCCTACTGAAGGTGGCAAGATCTTCGTAACTATCACTGAATATGCAGATCATGCTGCCAATGGTCGTGGTGAAGGTACTGTATTGGTTACCCGTAAAGCACTGTCTGTTACCCTGCCAAGCGGTGGTGCAGTGACTCTGAAGCCTGCTGATGTTGCTGCTGACGTTGGTGCTTCTATCACTGCTGGCCGTCAGGCTCGCTTCCTGTTTGAAGTTGAAACCAATCAGGGTGAAGTAGCTGTTAAGAAATCCAATGCTGAAGGCGTGGATATTCAGAATGGTACCCGCGGCACAGCACCGCTGGTAGATTTCACTCTGGGTTCAGGGGGTTCCGGTGGCGCCATGGTTGATACCTTATCAGGTTTATCAAGTGAGCAAGGTCAGTCCGGTGATATGACAATTGAAGAAGATAGTGCTACCCATATTAAATTCTCAAAACGTGATGAGGACGGCAAAGAGTTAGCTGGTGCAACTATGGAGTTGCGTGATTCATCTGGTAAAACTATTAGTACATGGATTTCAGATGGACAAGTGAAAGATTTCTACCTGTATCCAGGAAAATATACATTTGTCGAAACCGCAGCACCAGACGGTTATGAGGTAGCAACTGCTATTACCTTTACAGTTAATGAGCAAGGTCAGGTTACTGTAAATGGCAAAGCAACTAAAGGTGACGCTCATATT


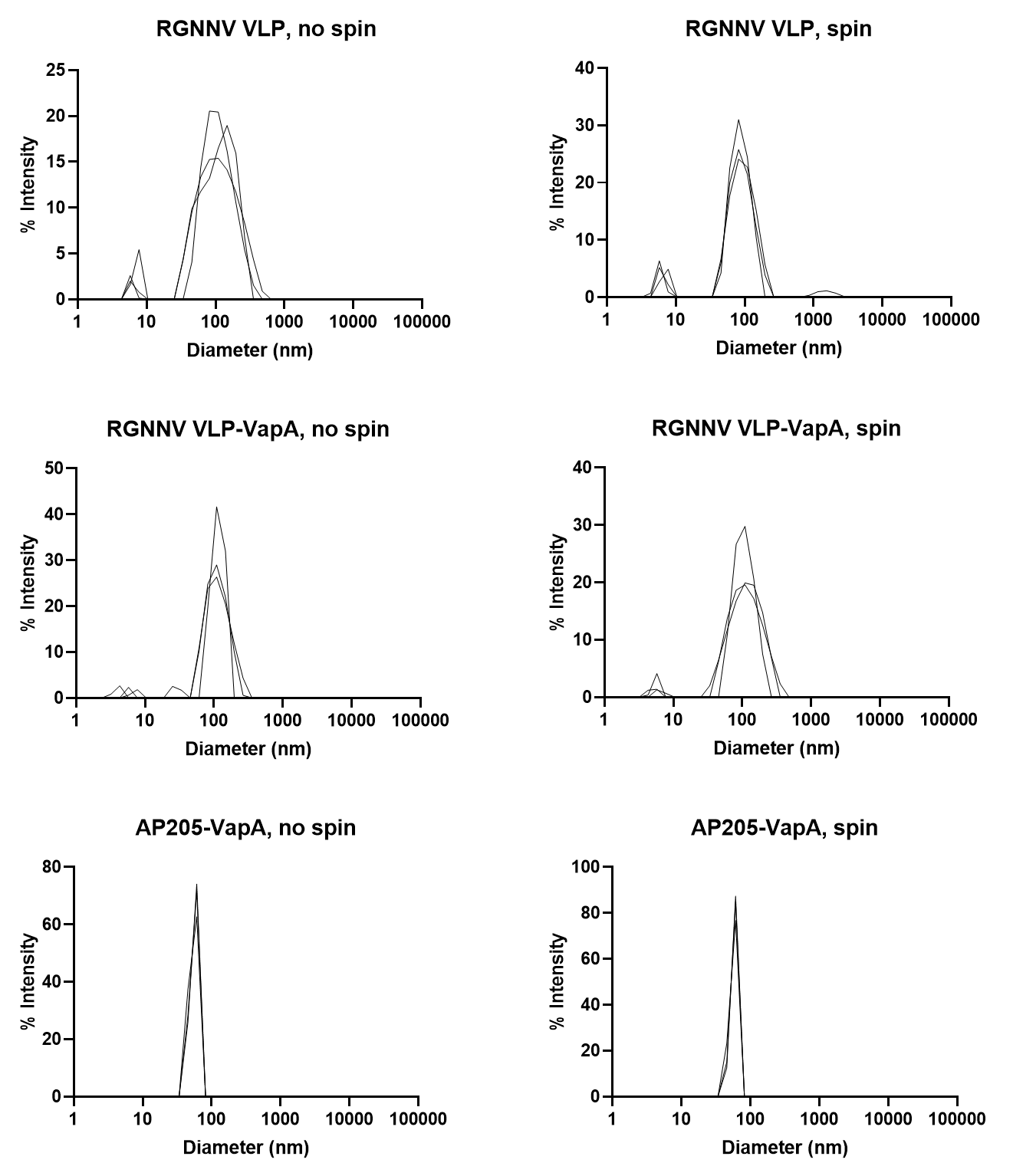


**Supplementary Figure 4.** Dynamic Light Scattering (DLS) analysis, showing a histogram of the % intensity of purified RGNNV VLP, RGNNV VLP-VapA and AP205-VapA pre-and post centrifugation (no spin, spin). The limited effect of centrifugation indicated that the VLPs did not aggregate following purification.

# Supplementary Tables

| **Vaccine** | **Dose**  **(100µl)** | **Tagging at vaccination** | **Fish**  **number** | **Tank no. at vaccination** | **Tagging at infection (Operculum clip)** |
| --- | --- | --- | --- | --- | --- |
| RGNNV VLP-VapA | 22µg |  | 100 | I | Right/Low |
| RGNNV VLP-VapA+FIA | 22µg | AD fin cut | 100 | II | Right/High |
| AP205 VLP-VapA | 17µg |  | 100 | III | Left/Low |
| AP205 VLP-VapA+FIA | 17µg |  | 100 | IV | Left/High |
| FKC | 10^6^ CFU | AD fin cut | 100 | III | Left/Low |
| FKC+FIA | 10^6^ CFU | AD fin cut | 100 | IV | Left/High |
| Buffer |  | AD fin cut | 100 | I | Right/Low |
| Buffer+FIA |  |  | 100 | II | Right/High |

**Supplementary Table 1.** Vaccination Trial-1. Experimental setup with 4 tanks each containing two groups of vaccinated fish (13±2g) distinguished by +/- clip of adipose fin. Individual group tagging by operculum clips before challenge allowed mixing of all 8 groups at the time of challenge. Vaccine doses specify amounts fusion proteins (Cp SpyTag-VapA SpyCatcher)

**Supplementary Table 2.** Vaccination Trial-1. Significance of difference between vaccine groups in terms of antibody response against vaccine antigens at 50 dpv. The Kruskal-Wallis test and the post hoc Dunn's multiple comparisons test were applied using GraphPad Prism version 8.3.0 (USA). (Kruskal WH, Wallis WA. Use of Ranks in One-Criterion Variance Analysis. *J Am Stat Assoc* (1952) 47:583–621. doi: 10.1080/01621459.1952.10483441)

| **Group comparison** | | | **Significant** | **Summary** | ***p*-value** |
| --- | --- | --- | --- | --- | --- |
| **Vaccine antigen: *A. salmonicida*** | | | | | |
| RGNNV VLP-VapA | vs | RGNNV VLP-VapA+FIA | No | ns | 0,4736 |
|  |  | AP205 VLP-VapA | No | ns | >0.9999 |
|  |  | AP205 VLP-VapA+FIA | No | ns | 0,3877 |
|  |  | FKC | Yes | *** | 0,0001 |
|  |  | FKC+FIA | Yes | **** | <0.0001 |
|  |  | Buffer | No | ns | >0.9999 |
|  |  | Buffer + FIA | No | ns | >0.9999 |
| RGNNV VLP-VapA+FIA | vs | AP205 VLP-VapA | No | ns | >0.9999 |
|  |  | AP205 VLP-VapA+FIA | No | ns | >0.9999 |
|  |  | FKC | No | ns | 0,7144 |
|  |  | FKC+FIA | No | ns | 0,4863 |
|  |  | Buffer | Yes | * | 0,0109 |
|  |  | Buffer + FIA | No | ns | >0.9999 |
| AP205 VLP-VapA | vs | AP205 VLP-VapA+FIA | No | ns | 0,8391 |
|  |  | FKC | Yes | *** | 0,0004 |
|  |  | FKC+FIA | Yes | *** | 0,0002 |
|  |  | Buffer | No | ns | >0.9999 |
|  |  | Buffer + FIA | No | ns | >0.9999 |
| AP205 VLP-VapA+FIA | vs | FKC | No | ns | 0,8598 |
|  |  | FKC+FIA | No | ns | 0,5908 |
|  |  | Buffer | Yes | ** | 0,0083 |
|  |  | Buffer + FIA | No | ns | >0.9999 |
| FKC | vs | FKC+FIA | No | ns | >0.9999 |
|  |  | Buffer | Yes | **** | <0.0001 |
|  |  | Buffer + FIA | Yes | ** | 0,0062 |
| FKC+FIA | vs | Buffer | Yes | **** | <0.0001 |
|  |  | Buffer + FIA | Yes | ** | 0,0035 |
| Buffer | vs | Buffer + FIA | No | ns | >0.9999 |
| **Vaccine antigen: VapA-SpyCatcher** | | |  |  |  |
| RGNNV VLP-VapA | vs | RGNNV VLP-VapA+FIA | No | ns | 0,2324 |
|  |  | AP205 VLP-VapA | No | ns | >0.9999 |
|  |  | AP205 VLP-VapA+FIA | No | ns | 0,142 |
|  |  | FKC | No | ns | >0.9999 |
|  |  | FKC+FIA | No | ns | >0.9999 |
|  |  | Buffer | No | ns | 0,1704 |
|  |  | Buffer+FIA | No | ns | >0.9999 |
| RGNNV VLP-VapA+FIA | vs | AP205 VLP-VapA | No | ns | 0,892 |
|  |  | AP205 VLP-VapA+FIA | No | ns | >0.9999 |
|  |  | FKC | Yes | *** | 0,0002 |
|  |  | FKC+FIA | No | ns | >0.9999 |
|  |  | Buffer | Yes | **** | <0.0001 |
|  |  | Buffer + FIA | Yes | *** | 0,0003 |
| AP205 VLP-VapA | vs | AP205 VLP-VapA+FIA | No | ns | 0,5864 |
|  |  | FKC | No | ns | 0,5278 |
|  |  | FKC+FIA | No | ns | >0.9999 |
|  |  | Buffer | Yes | * | 0,0339 |
|  |  | Buffer + FIA | No | ns | 0,6177 |
| AP205 VLP-VapA+FIA | vs | FKC | Yes | **** | <0.0001 |
|  |  | FKC+FIA | No | ns | >0.9999 |
|  |  | Buffer | Yes | **** | <0.0001 |
|  |  | Buffer + FIA | Yes | *** | 0,0001 |
| FKC | vs | FKC+FIA | Yes | * | 0,0176 |
|  |  | Buffer | No | ns | >0.9999 |
|  |  | Buffer + FIA | No | ns | >0.9999 |
| FKC+FIA | vs | Buffer | Yes | *** | 0,0005 |
|  |  | Buffer + FIA | Yes | * | 0,0218 |
| Buffer | vs | Buffer + FIA | No | ns | >0.9999 |
| **Vaccine antigen: LPS** | |  |  |  |  |
| RGNNV VLP-VapA | vs | RGNNV VLP-VapA+FIA | No | ns | >0.9999 |
|  |  | AP205 VLP-VapA | No | ns | >0.9999 |
|  |  | AP205 VLP-VapA+FIA | No | ns | 0,1649 |
|  |  | FKC | No | ns | 0,34 |
|  |  | FKC+FIA | Yes | * | 0,0185 |
|  |  | Buffer | No | ns | >0.9999 |
|  |  | Buffer + FIA | No | ns | >0.9999 |
| RGNNV VLP-VapA+FIA | vs | AP205 VLP-VapA | No | ns | >0.9999 |
|  |  | AP205 VLP-VapA+FIA | No | ns | >0.9999 |
|  |  | FKC | Yes | *** | 0,0001 |
|  |  | FKC+FIA | Yes | **** | <0.0001 |
|  |  | Buffer | No | ns | >0.9999 |
|  |  | Buffer + FIA | No | ns | 0,6309 |
| AP205 VLP-VapA | vs | AP205 VLP-VapA+FIA | No | ns | 0,1673 |
|  |  | FKC | No | ns | 0,3354 |
|  |  | FKC+FIA | Yes | * | 0,0182 |
|  |  | Buffer | No | ns | >0.9999 |
|  |  | Buffer + FIA | No | ns | >0.9999 |
| AP205 VLP-VapA+FIA | vs | FKC | Yes | **** | <0.0001 |
|  |  | FKC+FIA | Yes | **** | <0.0001 |
|  |  | Buffer | No | ns | 0,7994 |
|  |  | Buffer + FIA | No | ns | 0,0793 |
| FKC | vs | FKC+FIA | No | ns | >0.9999 |
|  |  | Buffer | No | ns | 0,0595 |
|  |  | Buffer + FIA | No | ns | 0,6389 |
| FKC+FIA | vs | Buffer | Yes | ** | 0,002 |
|  |  | Buffer + FIA | Yes | * | 0,0422 |
| Buffer | vs | Buffer + FIA | No | ns | >0.9999 |

Indication of Symbols: ns, non-significant; p, significance level; * *p*≤0.05; ** *p*≤0.01; *** *p*≤0.001

**Supplementary Table 3** Vaccination Trial-1. Significance of difference between vaccine groups in terms of antibody response at 69 dpv. The Kruskal-Wallis test and the post hoc Dunn's multiple comparisons test was applied as for data in Table 2.

| **Group comparison** | | | **Significant** | **Summary** | ***p*-value** |
| --- | --- | --- | --- | --- | --- |
| **Vaccine antigen: *A. salmonicida*** | | |  |  |  |
| RGNNV VLP-VapA | vs | RGNNV VLP-VapA+FIA | Yes | * | 0,0169 |
|  |  | AP205 VLP-VapA | No | ns | >0.9999 |
|  |  | AP205 VLP-VapA+FIA | Yes | ** | 0,0052 |
|  |  | FKC | Yes | **** | <0.0001 |
|  |  | FKC+FIA | Yes | **** | <0.0001 |
|  |  | Buffer | No | ns | >0.9999 |
|  |  | Buffer + FIA | No | ns | >0.9999 |
| RGNNV VLP-VapA+FIA | vs | AP205 VLP-VapA | No | ns | 0,1397 |
|  |  | AP205 VLP-VapA+FIA | No | ns | >0.9999 |
|  |  | FKC | No | ns | >0.9999 |
|  |  | FKC+FIA | No | ns | 0,1282 |
|  |  | Buffer | Yes | **** | <0.0001 |
|  |  | Buffer + FIA | No | ns | 0,5784 |
| AP205 VLP-VapA | vs | AP205 VLP-VapA+FIA | Yes | * | 0,0427 |
|  |  | FKC | Yes | *** | 0,0005 |
|  |  | FKC+FIA | Yes | **** | <0.0001 |
|  |  | Buffer | No | ns | 0,7694 |
|  |  | Buffer + FIA | No | ns | >0.9999 |
| AP205 VLP-VapA+FIA | vs | FKC | No | ns | >0.9999 |
|  |  | FKC+FIA | No | ns | >0.9999 |
|  |  | Buffer | Yes | **** | <0.0001 |
|  |  | Buffer + FIA | No | ns | 0,1831 |
| FKC | vs | FKC+FIA | No | ns | >0.9999 |
|  |  | Buffer | Yes | **** | <0.0001 |
|  |  | Buffer + FIA | Yes | ** | 0,0037 |
| FKC+FIA | vs | Buffer | Yes | **** | <0.0001 |
|  |  | Buffer + FIA | Yes | **** | <0.0001 |
| Buffer | vs | Buffer + FIA | No | ns | 0,2043 |
| **Vaccine antigen: VapA-SpyCatcher** | | |  |  |  |
| RGNNV VLP-VapA | vs | RGNNV VLP-VapA+FIA | Yes | *** | 0,0004 |
|  |  | AP205 VLP-VapA | No | ns | >0.9999 |
|  |  | AP205 VLP-VapA+FIA | No | ns | 0,1059 |
|  |  | FKC | No | ns | >0.9999 |
|  |  | FKC+FIA | No | ns | 0,9936 |
|  |  | Buffer | No | ns | 0,2158 |
|  |  | Buffer + FIA | No | ns | >0.9999 |
| RGNNV VLP-VapA+FIA | vs | AP205 VLP-VapA | Yes | ** | 0,0013 |
|  |  | AP205 VLP-VapA+FIA | No | ns | >0.9999 |
|  |  | FKC | Yes | **** | <0.0001 |
|  |  | FKC+FIA | No | ns | >0.9999 |
|  |  | Buffer | Yes | **** | <0.0001 |
|  |  | Buffer + FIA | Yes | **** | <0.0001 |
| AP205 VLP-VapA | vs | AP205 VLP-VapA+FIA | No | ns | 0,2495 |
|  |  | FKC | No | ns | >0.9999 |
|  |  | FKC+FIA | No | ns | >0.9999 |
|  |  | Buffer | Yes | * | 0,0464 |
|  |  | Buffer + FIA | No | ns | 0,3603 |
| AP205 VLP-VapA+FIA | vs | FKC | Yes | ** | 0,0059 |
|  |  | FKC+FIA | No | ns | >0.9999 |
|  |  | Buffer | Yes | **** | <0.0001 |
|  |  | Buffer + FIA | Yes | **** | <0.0001 |
| FKC | vs | FKC+FIA | No | ns | 0,0989 |
|  |  | Buffer | No | ns | >0.9999 |
|  |  | Buffer + FIA | No | ns | >0.9999 |
| FKC+FIA | vs | Buffer | Yes | **** | <0.0001 |
|  |  | Buffer + FIA | Yes | *** | 0,0009 |
| Buffer | vs | Buffer + FIA | No | ns | >0.9999 |
| **Vaccine antigen: LPS** | |  |  |  |  |
| RGNNV VLP-VapA | vs | RGNNV VLP-VapA+FIA | No | ns | >0.9999 |
|  |  | AP205 VLP-VapA | No | ns | >0.9999 |
|  |  | AP205 VLP-VapA+FIA | No | ns | >0.9999 |
|  |  | FKC | Yes | **** | <0.0001 |
|  |  | FKC+FIA | Yes | **** | <0.0001 |
|  |  | Buffer | No | ns | >0.9999 |
|  |  | Buffer + FIA | Yes | * | 0,0273 |
| RGNNV VLP-VapA+FIA | vs | AP205 VLP-VapA | No | ns | 0,8015 |
|  |  | AP205 VLP-VapA+FIA | No | ns | 0,2414 |
|  |  | FKC | Yes | **** | <0.0001 |
|  |  | FKC+FIA | Yes | **** | <0.0001 |
|  |  | Buffer | No | ns | 0,4264 |
|  |  | Buffer + FIA | Yes | *** | 0,0001 |
| AP205 VLP-VapA | vs | AP205 VLP-VapA+FIA | No | ns | >0.9999 |
|  |  | FKC | Yes | *** | 0,0003 |
|  |  | FKC+FIA | Yes | **** | <0.0001 |
|  |  | Buffer | No | ns | >0.9999 |
|  |  | Buffer + FIA | No | ns | 0,4331 |
| AP205 VLP-VapA+FIA | vs | FKC | Yes | * | 0,0123 |
|  |  | FKC+FIA | Yes | *** | 0,0007 |
|  |  | Buffer | No | ns | >0.9999 |
|  |  | Buffer + FIA | No | ns | >0.9999 |
| FKC | vs | FKC+FIA | No | ns | >0.9999 |
|  |  | Buffer | Yes | ** | 0,0012 |
|  |  | Buffer + FIA | No | ns | >0.9999 |
| FKC+FIA | vs | Buffer | Yes | **** | <0.0001 |
|  |  | Buffer + FIA | No | ns | 0,1251 |
| Buffer | vs | Buffer + FIA | No | ns | 0,981 |

Indication of Symbols: ns, non-significant; p, significance level; * *p*≤0.05; ** *p*≤0.01; *** *p*≤0.001

**Supplementary Table 4** Vaccination Trial-2. Significance of difference between vaccine groups in terms of antibody response at 48 dpv. The Kruskal-Wallis test was applied as for data in Table 2.

| **Group comparison** | | | **Significant** | **Summary** | ***p*-value** |
| --- | --- | --- | --- | --- | --- |
| **Vaccine antigen: *A. salmonicida*** | | |  |  |  |
| RGNNV VLP-VapA | vs | RGNNV VLP-VapA+FIA | No | ns | >0.9999 |
|  |  | RGNNV VLP+VapA | No | ns | >0.9999 |
|  |  | AP205 VLP-VapA | No | ns | >0.9999 |
|  |  | AP205 VLP-VapA+FIA | No | ns | >0.9999 |
|  |  | AP205 VLP+VapA | No | ns | >0.9999 |
|  |  | FKC | No | ns | >0.9999 |
|  |  | FKC+FIA | No | ns | >0.9999 |
|  |  | Buffer | No | ns | 0,2282 |
|  |  | Buffer + FIA | No | ns | >0.9999 |
| RGNNV VLP-VapA+FIA | vs | RGNNV VLP+VapA | No | ns | >0.9999 |
|  |  | AP205 VLP-VapA | No | ns | >0.9999 |
|  |  | AP205 VLP-VapA+FIA | No | ns | >0.9999 |
|  |  | AP205 VLP+VapA | No | ns | >0.9999 |
|  |  | FKC | No | ns | >0.9999 |
|  |  | FKC+FIA | No | ns | >0.9999 |
|  |  | Buffer | No | ns | 0,1055 |
|  |  | Buffer + FIA | No | ns | >0.9999 |
| RGNNV VLP+VapA | vs | AP205 VLP-VapA | No | ns | >0.9999 |
|  |  | AP205 VLP-VapA+FIA | No | ns | >0.9999 |
|  |  | AP205 VLP+VapA | No | ns | >0.9999 |
|  |  | FKC | Yes | * | 0,0164 |
|  |  | FKC+FIA | Yes | * | 0,0238 |
|  |  | Buffer | No | ns | >0.9999 |
|  |  | Buffer + FIA | No | ns | >0.9999 |
| AP205 VLP-VapA | vs | AP205 VLP-VapA+FIA | No | ns | >0.9999 |
|  |  | AP205 VLP+VapA | No | ns | 0,2058 |
|  |  | FKC | No | ns | >0.9999 |
|  |  | FKC+FIA | No | ns | >0.9999 |
|  |  | Buffer | Yes | * | 0,0127 |
|  |  | Buffer + FIA | No | ns | 0,2282 |
| AP205 VLP-VapA+FIA | vs | AP205 VLP+VapA | No | ns | >0.9999 |
|  |  | FKC | No | ns | >0.9999 |
|  |  | FKC+FIA | No | ns | >0.9999 |
|  |  | Buffer | No | ns | 0,1422 |
|  |  | Buffer + FIA | No | ns | >0.9999 |
| AP205 VLP+VapA | vs | FKC | Yes | ** | 0,0014 |
|  |  | FKC+FIA | Yes | ** | 0,0021 |
|  |  | Buffer | No | ns | >0.9999 |
|  |  | Buffer + FIA | No | ns | >0.9999 |
| FKC | vs | FKC+FIA | No | ns | >0.9999 |
|  |  | Buffer | Yes | **** | <0.0001 |
|  |  | Buffer + FIA | Yes | ** | 0,0016 |
| FKC+FIA | vs | Buffer | Yes | **** | <0.0001 |
|  |  | Buffer + FIA | Yes | ** | 0,0024 |
| Buffer | vs | Buffer + FIA | No | ns | >0.9999 |
| **Vaccine antigen: VapA-SpyCatcher** | | |  |  |  |
| RGNNV VLP-VapA | vs | RGNNV VLP-VapA+FIA | No | ns | >0.9999 |
|  |  | RGNNV VLP+VapA | No | ns | >0.9999 |
|  |  | AP205 VLP-VapA | No | ns | >0.9999 |
|  |  | AP205 VLP-VapA+FIA | No | ns | >0.9999 |
|  |  | AP205 VLP+VapA | No | ns | >0.9999 |
|  |  | FKC | No | ns | >0.9999 |
|  |  | FKC+FIA | No | ns | >0.9999 |
|  |  | Buffer | Yes | * | 0,0338 |
|  |  | Buffer + FIA | Yes | * | 0,0206 |
| RGNNV VLP-VapA+FIA | vs | RGNNV VLP+VapA | No | ns | >0.9999 |
|  |  | AP205 VLP-VapA | No | ns | >0.9999 |
|  |  | AP205 VLP-VapA+FIA | No | ns | >0.9999 |
|  |  | AP205 VLP+VapA | No | ns | 0,4086 |
|  |  | FKC | No | ns | 0,084 |
|  |  | FKC+FIA | No | ns | >0.9999 |
|  |  | Buffer | Yes | ** | 0,0011 |
|  |  | Buffer + FIA | Yes | *** | 0,0006 |
| RGNNV VLP+VapA | vs | AP205 VLP-VapA | No | ns | 0,8264 |
|  |  | AP205 VLP-VapA+FIA | No | ns | >0.9999 |
|  |  | AP205 VLP+VapA | No | ns | >0.9999 |
|  |  | FKC | No | ns | >0.9999 |
|  |  | FKC+FIA | No | ns | >0.9999 |
|  |  | Buffer | No | ns | >0.9999 |
|  |  | Buffer + FIA | No | ns | >0.9999 |
| AP205 VLP-VapA | vs | AP205 VLP-VapA+FIA | No | ns | >0.9999 |
|  |  | AP205 VLP+VapA | No | ns | 0,201 |
|  |  | FKC | Yes | * | 0,037 |
|  |  | FKC+FIA | No | ns | 0,5991 |
|  |  | Buffer | Yes | *** | 0,0004 |
|  |  | Buffer + FIA | Yes | *** | 0,0002 |
| AP205 VLP-VapA+FIA | vs | AP205 VLP+VapA | No | ns | 0,3525 |
|  |  | FKC | No | ns | 0,0708 |
|  |  | FKC+FIA | No | ns | 0,9876 |
|  |  | Buffer | Yes | *** | 0,0009 |
|  |  | Buffer + FIA | Yes | *** | 0,0005 |
| AP205 VLP+VapA | vs | FKC | No | ns | >0.9999 |
|  |  | FKC+FIA | No | ns | >0.9999 |
|  |  | Buffer | No | ns | >0.9999 |
|  |  | Buffer + FIA | No | ns | >0.9999 |
| FKC | vs | FKC+FIA | No | ns | >0.9999 |
|  |  | Buffer | No | ns | >0.9999 |
|  |  | Buffer + FIA | No | ns | >0.9999 |
| FKC+FIA | vs | Buffer | No | ns | >0.9999 |
|  |  | Buffer + FIA | No | ns | >0.9999 |
| Buffer | vs | Buffer + FIA | No | ns | >0.9999 |
| **Vaccine antigen: LPS** | |  |  |  |  |
| RGNNV VLP-VapA | vs | RGNNV VLP-VapA+FIA | No | ns | >0.9999 |
|  |  | RGNNV VLP+VapA | No | ns | >0.9999 |
|  |  | AP205 VLP-VapA | No | ns | >0.9999 |
|  |  | AP205 VLP-VapA+FIA | No | ns | 0,9453 |
|  |  | AP205 VLP+VapA | No | ns | >0.9999 |
|  |  | FKC | No | ns | >0.9999 |
|  |  | FKC+FIA | No | ns | >0.9999 |
|  |  | Buffer | No | ns | >0.9999 |
|  |  | Buffer + FIA | No | ns | >0.9999 |
| RGNNV VLP-VapA+FIA | vs | RGNNV VLP+VapA | No | ns | >0.9999 |
|  |  | AP205 VLP-VapA | No | ns | >0.9999 |
|  |  | AP205 VLP-VapA+FIA | No | ns | >0.9999 |
|  |  | AP205 VLP+VapA | No | ns | >0.9999 |
|  |  | FKC | Yes | * | 0,0213 |
|  |  | FKC+FIA | Yes | * | 0,0146 |
|  |  | Buffer | No | ns | >0.9999 |
|  |  | Buffer + FIA | No | ns | >0.9999 |
| RGNNV VLP+VapA | vs | AP205 VLP-VapA | No | ns | >0.9999 |
|  |  | AP205 VLP-VapA+FIA | No | ns | >0.9999 |
|  |  | AP205 VLP+VapA | No | ns | >0.9999 |
|  |  | FKC | Yes | * | 0,0369 |
|  |  | FKC+FIA | Yes | * | 0,0257 |
|  |  | Buffer | No | ns | >0.9999 |
|  |  | Buffer + FIA | No | ns | >0.9999 |
| AP205 VLP-VapA | vs | AP205 VLP-VapA+FIA | No | ns | >0.9999 |
|  |  | AP205 VLP+VapA | No | ns | >0.9999 |
|  |  | FKC | No | ns | 0,6299 |
|  |  | FKC+FIA | No | ns | 0,4752 |
|  |  | Buffer | No | ns | >0.9999 |
|  |  | Buffer + FIA | No | ns | >0.9999 |
| AP205 VLP-VapA+FIA | vs | AP205 VLP+VapA | No | ns | >0.9999 |
|  |  | FKC | Yes | ** | 0,0025 |
|  |  | FKC+FIA | Yes | ** | 0,0017 |
|  |  | Buffer | No | ns | >0.9999 |
|  |  | Buffer + FIA | No | ns | >0.9999 |
| AP205 VLP+VapA | vs | FKC | No | ns | 0,2768 |
|  |  | FKC+FIA | No | ns | 0,2035 |
|  |  | Buffer | No | ns | >0.9999 |
|  |  | Buffer + FIA | No | ns | >0.9999 |
| FKC | vs | FKC+FIA | No | ns | >0.9999 |
|  |  | Buffer | Yes | * | 0,022 |
|  |  | Buffer + FIA | No | ns | >0.9999 |
| FKC+FIA | vs | Buffer | Yes | * | 0,0151 |
|  |  | Buffer + FIA | No | ns | 0,8652 |
| Buffer | vs | Buffer + FIA | No | ns | >0.9999 |

Indication of Symbols: ns, non-significant; p, significance level; * *p*≤0.05; ** *p*≤0.01; *** *p*≤0.001
